# Silencing SFRP1 in bone mesenchymal stem cells alleviates pediatric B-ALL-driven bone loss by activating Wnt/β-catenin signaling

**DOI:** 10.1016/j.jot.2026.101071

**Published:** 2026-03-24

**Authors:** Mengxia Li, Xu He, Xingzhi Liu, Mimi Chen, Qian Sun, Ronghui Yu, Wendong Liu, Qi Wang, Guanghao Su, Qin Shi, Xiaodong Wang

**Affiliations:** a Department of Nephrology and Immunology, Children's Hospital of Soochow University, Suzhou, 215025, Jiangsu Province, China; b Department of Orthopedics, The First Affiliated Hospital of Soochow University, Orthopedic Institute of Soochow University, Suzhou, 215031, Jiangsu Province, China; c Central Laboratory, Ruijin Hospital Lu Wan Branch, Shanghai Jiao Tong University School of Medicine, Shanghai, 200020, China; d Department of Orthopedics, Children's Hospital of Soochow University, Suzhou, 215025, Jiangsu Province, China; e Department of Clinical Laboratory, Children's Hospital of Soochow University, Suzhou, 215025, Jiangsu Province, China

**Keywords:** Bone loss, Mesenchymal stem cells, Pediatric acute lymphoblastic leukemia SFRP1, shSFRP1@Lipo-E7

## Abstract

**Background:**

Bone loss is the most common skeletal complication of childhood acute lymphoblastic leukemia (ALL) and seriously affects the long-term survival quality of children. However, the mechanisms behind bone loss are complicated and need to be elucidated. This study seeks to examine the principal parameters influencing the osteogenic development of bone marrow mesenchymal stem cells (BMSCs) in pediatric patients with B-cell acute lymphoblastic leukemia (B-ALL) experiencing bone loss, and to identify viable ways for alleviating bone loss.

**Methods:**

Firstly, bone mass of adolescent B-ALL patients and mice were evaluated with aged-matched cohorts. Then, human BMSCs (hBMSCs) were isolated from pediatric B-ALL patients and characterized by Flow Cytometry Assay (FCA). ALP, ARS, Oil Red O and toluidine blue staining were used to evaluate the trilineage differentiation of hBMSCs. Integrated RNA-seq and proteomic analyses were employed to identify differentially target osteogenic regulators in B-ALL-derived hBMSCs. On the based that secreted frizzled-related protein 1 (SFRP1) was demonstrated to be a regulator on B-ALL BMSC osteogenesis, BMSC-targeted liposomal nanocarriers were engineered to encapsulate lentiviral particles carrying therapeutic shSFRP1 RNA and grafted with E7 peptide (shSFRP1@Lipo-E7), enabling cell-specific gene silencing in BMSCs. Subsequently, shSFRP1@Lipo-E7 was delivered into B-ALL mice by tail vein injection and bone quality were evaluated by mico-CT and histomorphometric analysis.

**Results:**

Adolescent B-ALL patients exhibited significant vertebral bone loss, with 33.8% of patients affected. In B-ALL mice, BMD and newbone formation were markedly reduced, while osteoclast activity increased. An increased expression of SFRP1 was identified to impair osteogenesis of BMSCs from B-ALL patients. Consequently, a BMSC-targeted nanoplatform, E7 peptide-modified liposomes containing lentiviral shSFRP1 (shSFRP1@Lipo-E7) was constructed successfully. As anticipated, shSFRP1@Lipo-E7 effectively suppressed SFRP1 expression in trabecular osteoblasts and rescued B-ALL mice from bone loss, supported by significantly increasing BMD and improved trabecular structure. Both *in vitro* and vivo studies evidenced that silencing of SFRP1 activated Wnt/β-catenin signaling to promote BMSC osteogenesis and bone formation in B-ALL mice.

**Conclusion:**

High SFRP1 expression in B-ALL BMSCs suppresses osteogenesis and contributes to bone loss in B-ALL cohorts by inhibiting Wnt/β-catenin signaling, which afford a potential translatable target to reprogram bone homeostasis and prevent bone fragility in ALL patients.

**The Translational Potential of this Article:**

Given the negative correlation between SFRP1 overexpression in BMSCs of pediatric B-ALL related bone mass loss, precisely targeting SFRP1 in MSCs for intervention holds promise as a therapeutic strategy to rescue bone loss in this disease category.

## Graphical abstract

**Figure:**
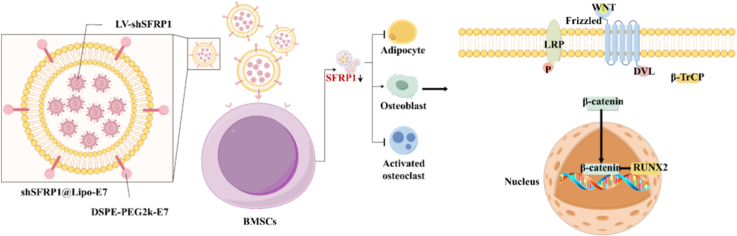

## 1 Introduction

Acute lymphoblastic leukemia (ALL), the most common pediatric malignancy, has achieved remarkable therapeutic progress, with 5-year survival rates exceeding 90% due to improved chemotherapy and immunotherapy regimens [1]. However, this success is accompanied by serious long-term complications, particularly skeletal morbidities such as osteoporosis (OP) and vertebral fractures, which affect 30–40% of survivors and may persist into adulthood [2,3]. Bone loss in ALL results from two converging mechanisms: direct leukemic infiltration that disrupts bone remodeling, and chemotherapy-induced toxicity—especially from glucocorticoids—which suppress osteoblast (OB) activity and enhance osteoclastogenesis [4,5]. Notably, up to 40% of pediatric ALL patients show reduced bone mineral density (BMD) at diagnosis, indicating that leukemia itself serves as an independent driver of skeletal pathology [6]. However, compared to tumor control, the mechanisms underlying ALL-associated bone loss have long lacked systematic elucidation, resulting in clinical management that remains largely empirical rather than driven by mechanistic interventions. Therefore, this study will focus on elucidating the key molecular mechanisms of bone loss in the ALL context to provide a basis for targeted interventions.

As multipotent stromal progenitors, BMSCs play a fundamental role in maintaining bone homeostasis through differentiation into osteoblasts and balancing osteoclast activity [7]. Under physiological conditions, several signaling pathways are involved in BMSC osteogenesis, including canonical Wnt/β-catenin, BMP/Smad, and Notch, etc [8,9]. However, the properties of BMSCs change under pathological conditions. For example, in OP, BMSCs exhibit impaired osteogenic differentiation, disrupting the coupling of bone formation and resorption and ultimately resulting in decreased bone mass and skeletal fragility [10]. BMSCs derived from systemic lupus erythematosus patients display aberrant upregulation of interferon-β, an inflammatory mediator that alters immunomodulatory function and suppresses osteogenic commitment [11]. Notably, bone loss is not exclusive to chronic metabolic or inflammatory diseases. In rapidly progressing hematologic malignancies such as acute myeloid leukemia (AML) and acute lymphoblastic leukemia (ALL), decreased bone mass, bone pain, and elevated fracture risk are equally common complications for which targeted clinical solutions remain lacking. Malignant infiltration by leukemic cells can reprogram BMSCs, suppressing their osteogenic differentiation and disrupting the coupled homeostasis of bone formation and resorption, ultimately accelerating bone loss [12]. Comparable mechanisms may operate in B-ALL, yet this remains largely unexplored, representing a crucial gap given the lymphoid origin of B-ALL and its distinct interactions with the bone marrow niche.

Secreted frizzled-related protein 1 (SFRP1) is a soluble antagonist of the Wnt signaling pathway that acts by sequestering Wnt ligands or competitively binding Frizzled receptors, thereby preventing canonical β-catenin activation. Given the essential role of Wnt/β-catenin signaling in osteoblast differentiation and bone formation, SFRP1 functions as a key regulator of skeletal homeostasis [13]. Under pathological conditions, aberrant SFRP1 expression has been implicated in several bone disorders. In age-related and postmenopausal OP, elevated SFRP1 expression in BMSCs disrupts the osteogenic-adipogenic balance, suppressing osteoblast maturation and promoting adipocyte differentiation, thereby accelerating trabecular bone loss [14]. While SFRP1 is often epigenetically silenced in solid tumors and myeloid malignancies such as acute myeloid leukemia, chronic lymphocytic leukemia, and multiple myeloma [15,16], its role in B-ALL-induced bone loss remains poorly defined. Although leukemia-associated bone loss is generally attributed to excessive osteoclast activation via the RANKL pathway [6], the contribution of SFRP1 to impaired osteogenesis warrants further investigation. Considering that B-ALL accounts for nearly 85% of pediatric leukemia cases, elucidating the functional impact of SFRP1 on BMSC behavior and niche remodeling could uncover novel mechanisms of leukemia-associated bone pathology and identify promising therapeutic targets.

During childhood growth and development, bone formation is more active, making bone mass accumulation more dependent on BMSC-mediated osteogenic differentiation. Current treatment strategies for leukemia-associated bone loss—such as bisphosphonates and denosumab—primarily inhibit osteoclast activity but have limited effects on osteogenesis. Moreover, these agents carry risks, including hypocalcemia, nephrotoxicity, and atypical femoral fractures in pediatric populations, restricting their long-term use [17,18]. Osteoanabolic therapies like teriparatide, which stimulate bone formation, are contraindicated in pediatric oncology due to potential concerns about promoting residual leukemic cell proliferation [19]. These limitations underscore the urgent need for approaches that can selectively enhance the osteogenic capacity of BMSCs to restore balanced bone remodeling while minimizing leukemia progression risks. Achieving this requires identifying novel molecular regulators of BMSC function within the leukemic microenvironment and developing efficient delivery systems capable of modulating these targets precisely in situ.

Lipid-based nanoparticle platforms, particularly liposomes, offer distinct advantages in this context owing to their high drug-loading capacity, tunable surface modifications for cell-specific targeting, and excellent biocompatibility [20]. Our group has previously developed liposome-based delivery systems capable of modulating disease-associated microenvironments, as exemplified by a folate-modified liposomal hydrogel that simultaneously targeted pro-inflammatory macrophages and restored cartilage lubrication in osteoarthritis rats, thereby addressing both biomechanical and inflammatory aspects of tissue degeneration [21]. These findings provide a technical foundation for extending liposomal delivery strategies to the regulation of BMSC function in leukemia-associated bone disorders. Consequently, liposome-based delivery represents a promising approach to precisely modulate the leukemic bone marrow microenvironment, restore osteogenic balance, and ultimately alleviate leukemia-induced bone loss.

In this study, elevated expression of SFRP1 was first identified as a factor inducing bone loss in the pediatric B-ALL cohorts. A targeted therapeutic strategy was subsequently developed to restore bone formation in leukemia-associated bone disease. In recent years, E7 peptide (EPLQLKM) has garnered significant attention due to its specific affinity for bone marrow mesenchymal stem cells (BMSCs). Integrating E7 peptide into materials enables selective capture and stable adhesion of BMSCs on material surfaces, thereby enhancing recruitment efficiency within bone tissue [22]. Compared to non-specific adhesion strategies, the E7-mediated recognition process offers greater targeting specificity. This helps reduce interference from irrelevant cells occupying the space and enhances the predictability of constructing the regenerative microenvironment. A liposome (Lipo) functionalized with the E7 was engineered to selectively deliver lentiviral vectors encoding SFRP1-targeting shRNA (shSFRP1@Lipo-E7) to BMSCs [23,24]. In leukemic mouse models, treatment with shSFRP1@Lipo-E7 efficiently silenced SFRP1 expression, reactivated canonical Wnt signaling, and restored trabecular bone mass. By investigating the abnormal differentiation patterns and intervention mechanisms of BMSCs within the malignant bone marrow microenvironment of the pediatric B-ALL, this study provides a potentially translatable strategy for managing leukemia-related bone complications from an osteogenic perspective.

## 2 Materials and methods

### 2.1 Ethics statement

This retrospective clinical study was approved by the Medical Ethics Committee of Children’s Hospital of Soochow University (No. 2024CS015) and conducted in accordance with the Declaration of Helsinki. All the pediatric patient data were anonymized to ensure complete privacy protection. Bone marrow (BM) specimens used for hBMSC extraction were obtained from leftover samples following diagnostic testing in the hospital's Laboratory Department, without causing additional discomfort to the children.

For animal experiments, 6-week-old female NOD-SCID gamma (NSG) mice were purchased from the Shanghai Model Organisms Center and housed under specific pathogen-free (SPF) conditions at Soochow University. All animal procedures were approved by the Experimental Animal Ethics Committee of Soochow University (No. SUDA20230625A02).

### 2.2 Computed tomography (CT) scanning of the pediatric B-ALL patients

In CT evaluation, the Hounsfield Unit (HU) serves as the fundamental quantitative scale defining tissue radiodensity. In bone, higher mineral content corresponds to greater density and thus higher HU values. Current evidence supports the establishment of threshold HU values from CT measurements of trabecular bone between T12 and L5 vertebrae for osteoporosis diagnosis [25], with T12 and L1 values emphasized in this study. Vertebral HU measurements at T12 or L1 were performed in children aged ≥6 years (yrs) with newly diagnosed the pediatric B-ALL prior to treatment between January 2020 and June 2023. A control group of 207 age- and sex-matched children (82 cases of traffic accident injuries, 38 from falls, and 87 with acute abdominal surgical conditions) who underwent thoracic or abdominal CT scans at Children's Hospital of Soochow University served as controls (Supplementary Table 1).

For each subject, trabecular bone from the T12 or L1 vertebra was measured using three standardized axial slices—superior, middle, and inferior vertebral body levels (Supplementary Fig. 1A). Elliptical regions of interest (ROIs) were manually placed in the anterior trabecular region, avoiding cortical bone and imaging artifacts. Measurements were performed with Neusoft PACS/RIS software (Neusoft Corporation), and mean HU values were recorded. The average of the three slices was defined as the final vertebral CT value [26].

### 2.3 Establishment of humanized B-ALL in mice

B-ALL mice were generated following a previously described method [6]. In brief, human pediatric B-cell acute lymphoblastic leukemia-1 (hB-ALL1, CL-0587, Wuhan Pricella Biotechnology Co., Ltd.) cells were cultured and prepared as a single-cell suspension in phosphate-buffered saline (PBS) before transplantation. For xenotransplantation, 1 × 10^6^ cells were intravenously injected into immunodeficient NSG mice via the tail vein. Peripheral blood was collected 7 days post-inoculation, and leukemic engraftment was verified through flow cytometry assay (FCA) using the human-specific surface marker CD45. Successful establishment of humanized B-ALL mice (mB-ALL) was defined as >1% human CD45^+^ cells in peripheral blood.

### 2.4 Micro-CT scanning of B-ALL mice

Twenty-eight days after xenotransplantation, femurs from control NSG (mCTR) and mB-ALL mice were analyzed in a high-resolution micro-computed tomography (micro-CT) system (Skyscan 1176, Bruker, Belgium), following previously published procedures [20]. Recorded parameters included bone mineral density (BMD), bone volume (BV), tissue volume (TV), bone volume fraction (BV/TV), trabecular number (Tb.N, mm^−1^), trabecular thickness (Tb.Th, mm), trabecular separation (Tb.Sp, mm), and structure model index (SMI).

### 2.5 Enzyme-linked immunosorbent assay (ELISA)

Serum samples from mB-ALL mice were collected 28 days after model establishment. The concentrations of procollagen type I N-terminal propeptide (PINP) and C-terminal telopeptide of type I collagen (CTX-I) were quantified using commercial ELISA kits (Elabscience, China) according to the manufacturer's instructions. Optical density was measured at 450 nm through the use of a SpectraMax M5 microplate reader (BioTek, USA).

### 2.6 Histological staining

Femoral specimens were fixed, decalcified, and embedded in paraffin. Serial 5 μm sections were prepared and stained with hematoxylin and eosin (H&E). Tartrate-resistant acid phosphatase (TRAP) staining was performed to identify and quantify osteoclasts, while Masson's trichrome staining was conducted to assess collagen deposition. Selected sections were used for immunohistochemistry (IHC) to detect the expression of the osteogenic marker *RUNX2* (Servicebio, China) and the Wnt signaling marker β-catenin (Servicebio, China), and immunofluorescence (IF) to detect the expression of the SFRP1 (Proteintech, China). Quantitative image analysis was conducted employing ImageJ software (Rawak Software Inc., USA).

### 2.7 BMSC isolation and culture

Human BM aspirates were cultured in α-MEM complete medium supplemented with 12.5% fetal bovine serum (FBS) according to the whole marrow adherence method [27]. The medium was changed twice weekly to promote the expansion of human BMSCs (hBMSCs). A phase-contrast microscope was used to document cell morphology at days 4, 7, and 10. Mouse BMSCs (mBMSCs) were isolated from the femurs and tibias of 6–8-week-old NSG mice [28,29]. All BMSCs used in the present study were at the P3-P5 passage stage.

### 2.8 Flow cytometry assay (FCA)

Third-passage hBMSCs were incubated with fluorochrome-conjugated antibodies against human CD44 (BioLegend, USA), CD90 (BioLegend, USA), CD34 (BioLegend, USA), and CD45 (BioLegend, USA). Flow cytometric analysis was performed with appropriate compensation controls, and data were processed with FlowJo software. The MSC phenotype was validated according to ISCT criteria [30] (CD44^+^/CD90^+^/CD34^–^/CD45^–^). mBMSCs were trypsinized, stained with fluorochrome-conjugated antibodies against mouse CD90, CD29, CD34, and CD45 (all from BioLegend, USA), and analyzed similarly.

### 2.9 Cell cycle analysis of the pediatric B-ALL-derived hBMSCs

Human BMSCs from healthy donors (hCTR) and B-ALL patients (hB-ALL) were seeded in 6-well plates (5 × 10^4^ cells/well) and serum-starved for 24 h before analysis. Cells were fixed in ethanol overnight at 4 °C, stained with PI/RNase A solution for 30 min at 37 °C in the dark, and filtered through 40 μm strainers. Cell cycle distribution was analyzed, and results were quantified with ModFit LT 5.0 software employing debris subtraction and doublet discrimination.

### 2.10 Cell proliferation assay

The pediatric hBMSCs from hCTR and hB-ALL groups were seeded in 96-well plates (2 × 10^3^ cells/well). CCK-8 reagent (Japan, Dojindo, CK04) was added at 0, 2, 4, 6, and 8 days post-seeding, followed by 2 h incubation. Absorbance at 450 nm was measured using a SpectraMax M5 microplate reader, and growth curves were generated based on OD values.

### 2.11 Trilineage differentiation of hBMSCs

The osteogenic, chondrogenic, and adipogenic differentiation potentials of hBMSCs were evaluated [31]. Media were changed every 3 days until differentiation was complete.

For osteogenic induction, BMSCs were cultured in osteogenic medium. After 7 days, cells were fixed with 4% paraformaldehyde (PFA) and stained with BCIP/NBT solution under light-protected conditions. The ImageJ was utilized for ALP-positive areas. After 21 days, cells were fixed in 70% ethanol and stained with 2% alizarin red S (pH 4.2) for 30 min at room temperature to detect calcium deposits. Quantitative analysis was performed with ImageJ.

For chondrogenic differentiation, cells were cultured in DMEM-HG supplemented with 10% FBS, 10 ng/mL transforming growth factor β1 (TGF-β1), 0.5 μg/mL insulin, and 50 μM ascorbic acid. On day 14, unfixed cells were stained with 0.1% toluidine blue (pH 2.5) for 15 min at room temperature, followed by aqueous intensification to visualize sulfated proteoglycans.

For adipogenic differentiation, after 21 days of induction, cells were fixed with 4% PFA and stained with 0.5% Oil Red O in 60% isopropanol at 37 °C for 20 min to visualize lipid droplets. Images were captured by microscopy, and quantification of all lineages was conducted.

### 2.12 Quantitative real-time PCR (qRT-PCR) analysis

Seven days after osteogenic or chondrogenic induction, total RNA was extracted by TRIzol reagent and reverse-transcribed into cDNAt. qRT-PCR was performed with Brilliant SYBR Green QPCR Master Mix (Takara, Japan). Relative expression levels were normalized to *GAPDH*, and the 2^–ΔΔCT^ method was used to calculate target gene expression. Primer sequences are listed in Supplementary Table 2.

### 2.13 Western blot (WB)

After 10 days of osteogenic or chondrogenic induction, or 14 days of adipogenic induction, proteins were extracted from cells. Protein concentrations were measured using the BCA assay (Beyotime, China). Equal amounts of 20 μg protein were separated by SDS-PAGE and transferred to PVDF membranes. Membranes were incubated with primary and secondary antibodies, and chemiluminescence was detected through ECL reagent. Band intensities were quantified with ImageJ and normalized to loading controls (GAPDH, Tubulin). Antibodies used are listed in Supplementary Table 3.

### 2.14 Multi-omics analysis

hBMSCs from five healthy pediatric donors and three age- and sex-matched the pediatric B-ALL patients were collected and stored in TRIzol at −80 °C before proteomic and transcriptomic profiling. Proteomics were conducted with LC-MS/MS (Q Exactive HF-X), and RNA-seq by Illumina NovaSeq 600). Basic information on BM samples used for multi-omics sequencing is provided in Supplementary Table 4.

### 2.15 The expression of β-catenin

mBMSCs derived from B-ALL mice were induced toward osteogenesis with recombinant SFRP1 protein (50 ng/mL) and/or SFRP1 inhibitor WAY-316606 (2 μM). After 7 days, total proteins were extracted, and expression levels of β-catenin and RUNX2 under different conditions were assessed by western blot.

### 2.16 Construction of shSFRP1 lentivirus

To effectively silence *SFRP1*, lentiviral vectors encoding SFRP1-specific short hairpin RNA (shSFRP1, 5′–3′ GCGAGTTTGCACTGAGGATGA, GenePharma, China) and GFP tag were constructed.

### 2.17 Construction and characterization of shSFRP1@Lipo-E7

Liposomes were prepared under the thin-film hydration method as described previously [21]. Briefly, 20 mg hydrogenated soybean phosphatidylcholine, 5 mg cholesterol, 2 mg stearylamine, and 2.76 mg DSPE-PEG_2_k-E7 (TransTech Biotechnology, China) were dissolved in 5 mL chloroform to form a uniform mixture. The solvent was evaporated to form a lipid film, which was hydrated with ddH_2_O and shSFRP1 lentivirus, followed by 30 min sonication and 0.2 μm extrusion to obtain shSFRP1-loaded liposomes (shSFRP1@Lipo-E7).

Transmission electron microscopy (TEM, JEOL JEM-1400, Japan) was employed to observe liposome morphology. Particle size and zeta potential were measured via a Malvern Zetasizer Pro (UK).

### 2.18 shSFRP1@Lipo-E7 uptake analysis

To evaluate the cellular uptake of liposomes, mBMSCs and hB-ALL1 cells (2 × 10^4^/well) were incubated with DiO-labeled liposomes (shSFRP1@Lipo-E7, Lipo, or Lipo-E7) for 12 and 24 h, respectively. Cells were washed with PBS, fixed with 4% PFA, and counterstained with DAPI. Fluorescence images were acquired by applying a confocal microscope (Leica, Germany). Quantitative analysis of liposome uptake was performed by calculating the DiO^+^/DAPI^+^ cell ratio from triplicate fields.

### 2.19 Administration of shSFRP1@Lipo-E7 in B-ALL mice

One week after model establishment, B-ALL mice were randomly assigned to seven groups: mB-ALL + PBS (treated with PBS), mB-ALL + Lipo (treated with Lipo), mB-ALL + Lipo-E7 (treated with Lipo-E7), mB-ALL + NC (treated with free LV-NC), mB-ALL + shSFRP1@Lipo-E7 (treated with shSFRP1@Lipo-E7), mB-ALL + NC@Lipo-E7 (treated with LV-NC@Lipo-E7), and mB-ALL + shSFRP1 (treated with free LV-shSFRP1). Mice in each group received 100 μL of the respective formulation via tail vein injection. After 3 weeks of continuous treatment, the mice were euthanized, and femurs, sera, and major organs were collected for subsequent analyses.

### 2.20 Immunofluorescence staining

Paraffin-embedded femoral sections underwent antigen retrieval, blocking with 3% BSA, and incubation with anti-SFRP1 antibody (Proteintech, 26460-1-AP, 1:200) for 12 h, followed by Alexa Fluor 594-conjugated secondary antibody (Abcam, ab150080, 1:500). Autofluorescence was quenched using TrueVIEW reagent (Vector Labs, USA), and nuclei were counterstained with DAPI for 10 min. Sections were mounted with ProLong Gold and visualized by a confocal microscope. Quantitative image analysis was conducted through ImageJ software.

### 2.21 Statistical analysis

Data were expressed as mean ± standard deviation (SD). Statistical analyses were conducted by unpaired Student's t-test (after confirming normality via Shapiro–Wilk test and homogeneity of variance via Levene's test) or one-way ANOVA followed by Tukey's post hoc test for multiple group comparisons. Categorical variables were analyzed with the chi-square test by means of Yates' continuity correction. All analyses were carried out in GraphPad Prism 9.0.0 (GraphPad Software, USA). A *P* value < 0.05 was considered statistically significant.

## 3 Results

### 3.1 Bone loss in the pediatric B-ALL patients

**Fig. 1:**
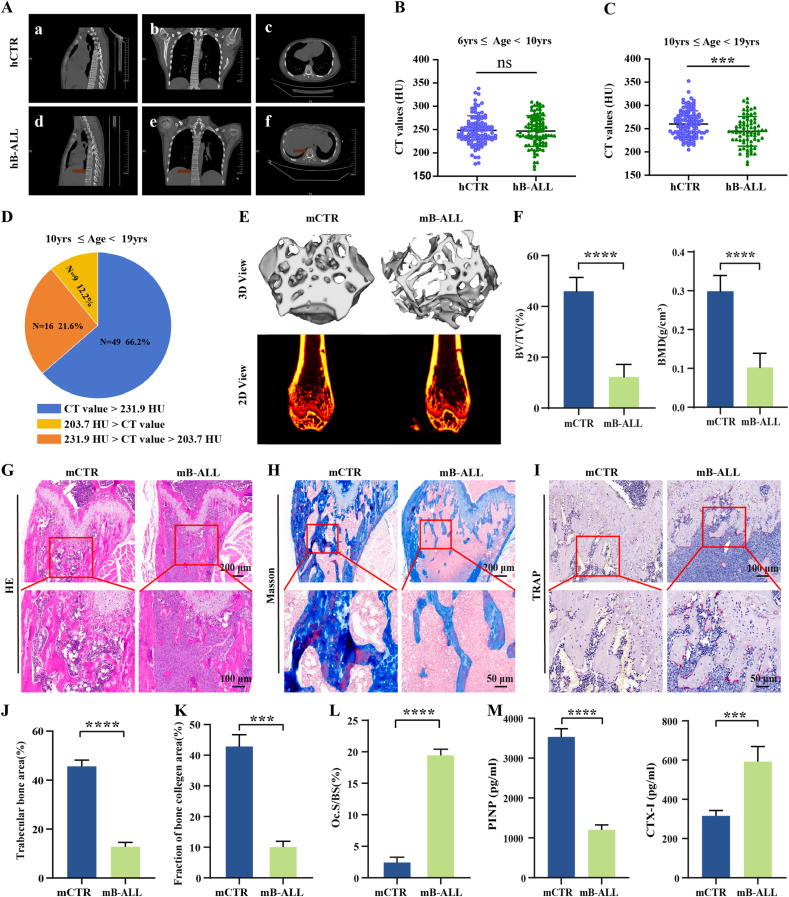
Bone loss in the pediatric B-ALL patients and B-ALL mice. (A) Representative thoracic vertebral CT scans of B-ALL patients with osteopenia or vertebral fractures (d-f) and healthy controls (hCTR, a-c). Red arrows indicate worm-eaten osteolytic lesions (d-f). (B-C) Comparison of vertebral CT values in B-ALL patients and health cohorts. (D) Distribution pattern of vertebral CT values in B-ALL patients aged 10–19 years. (E) 3D (top) and 2D (bottom) reconstructions of murine trabecular architecture obtained from micro-CT scans. (F) Quantitative analysis of murine femoral bone parameters, including BV/TV and BMD (n = 6). (G) H&E staining and (J) quantification of murine trabecular bone area (n = 6). (H) Masson's trichrome staining (blue, nascent collagen; red, mature bone) and (K) quantification of collagen volume fraction (n = 6). (I) TRAP staining of osteoclasts (red) in murine femoral sections and (L) quantification of Oc.S/BS (osteoclast surface per bone surface) (n = 6). (M) Serum levels of PINP and CTX-I in B-ALL mice (n = 6).; ∗∗∗*P* < 0.001, ∗∗∗∗*P* < 0.0001, ns: no significance.

Initial clinical observations revealed apparent bone loss in pediatric patients with B-ALL compared to healthy controls (hCTR) (Fig. 1A). To further quantify this, vertebral CT values were analyzed in pediatric B-ALL patients aged 6 to <19 years. Demographic characteristics showed no significant differences in age, sex, or BMI between the hB-ALL and hCTR cohorts (Supplementary Table 1). Comparative analysis of T12–L1 vertebral CT values indicated no significant reduction in B-ALL patients aged 6 to <10 years compared with matched controls (Fig. 1B). However, in the 10 to <19-year subgroup, vertebral CT values in B-ALL patients (244.4 ± 31.7 HU) were significantly lower than those in hCTR individuals (260.1 ± 28.2 HU) (Fig. 1C). Further stratification revealed that 33.8% of older hB-ALL patients had vertebral CT values below 231.9 HU, suggesting reduced bone mass, and 12.2% had values below 203.7 HU, meeting diagnostic criteria for osteoporosis (OP) (Fig. 1D). One patient also presented a vertebral compression fracture (Fig. 1A).

### 3.2 Bone loss in B-ALL mice

At 7 days post-induction, human CD45^+^ cells constituted 9.14 ± 0.46% of peripheral blood leukocytes in B-ALL mice, exceeding the diagnostic threshold of 0.01% (Supplementary Fig. 1B). Compared with healthy controls (mCTR), B-ALL mice exhibited marked splenomegaly, characterized by significantly increased spleen size and weight (Supplementary Fig. 1C). H&E staining further confirmed extensive leukemic infiltration in bone marrow (BM) cavities and spleen parenchyma, with diffuse sheets of immature, blast-like cells replacing normal hematopoietic elements (Supplementary Fig. 1D), indicating successful and stable model establishment.

After 4 weeks, micro-CT scanning of distal femurs revealed disorganized trabeculae, severe cancellous bone loss, and enlarged marrow cavities in mB-ALL mice compared to mCTR (Fig. 1E). Quantitative analysis showed significantly reduced BV/TV (12.28 ± 4.91% vs 45.96 ± 5.46%), BMD (0.103 ± 0.036 g/cm^3^ vs 0.299 ± 0.041 g/cm^3^), Tb.N (1.42 ± 0.46 mm^−1^ vs 3.59 ± 0.12 mm^−1^), and Tb.Th (0.085 ± 0.009 mm vs 0.128 ± 0.013 mm), along with elevated SMI (2.48 ± 0.28 vs 0.73 ± 0.22) and Tb.Sp (0.366 ± 0.067 mm vs 0.200 ± 0.020 mm) (Fig. 1F and Supplementary Fig. 1E). Histologically, H&E staining revealed a pronounced reduction in trabecular bone area (12.70 ± 1.85% vs 45.62 ± 3.81%) distal to the growth plate (Fig. 1G–J), while Masson's trichrome staining demonstrated decreased collagen deposition (11.42 ± 1.94% vs 43.48 ± 4.11%) (Fig. 1H–K). TRAP staining showed significantly increased osteoclast activity in B-ALL mice (Oc.S/BS: 2.60 ± 0.66% vs 19.61 ± 0.79%) (Fig. 1I–L), implicating osteoclast-mediated bone resorption as a major contributor to bone degradation.

Serum biomarker analysis revealed markedly reduced PINP (1202.5 ± 123.7 pg/mL vs 3531 ± 207.9 pg/mL) and elevated CTX-I (592.2 ± 76.9 pg/mL vs 315.3 ± 27.6 pg/mL) levels in B-ALL mice (Fig. 1M), indicating suppressed bone formation and enhanced resorption. Although both impaired osteogenesis and increased osteoclast activity contributed to leukemia-associated bone loss, the 1.94-fold decrease in PINP and 0.88-fold increase in CTX-I suggested that impaired osteogenesis played a dominant role.

In summary, consistent with the findings in B-ALL patients, pronounced bone loss was also observed in humanized B-ALL mice. This provided a solid foundation for further elucidating the molecular mechanisms of leukemia-induced bone loss and developing targeted intervention strategies with the established B-ALL mouse model.

### 3.3 Leukemia-associated hBMSCs display impaired osteogenic capacity

Primary hBMSCs derived from hCTR samples exhibited typical spindle- or stellate-shaped morphology with abundant cytoplasm. On day 4 of culture, elongated adherent cells appeared, followed by colony formation on day 7. By day 10, dense colonies developed, forming confluent monolayers with characteristic whorled arrangements after passaging (Supplementary Fig. 2A). Flow cytometric analysis displayed low or no expression of hematopoietic markers CD34 and CD45 (0.69% and 0%, respectively), alongside high expression of mesenchymal markers CD90 and CD44 (95.9% and 99.8%, respectively) (Supplementary Fig. 2B), validating hBMSC purity. Trilineage differentiation assays demonstrated successful differentiation into osteogenic, adipogenic, and chondrogenic lineages (Supplementary Fig. 2C).

**Fig. 2:**
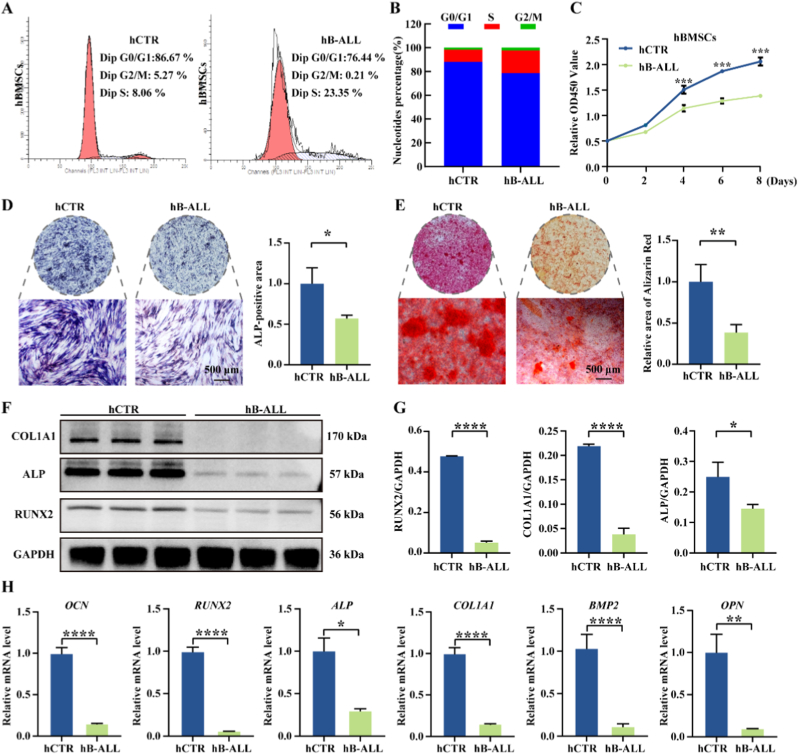
Functional impairment of the pediatric hB-ALL BMSCs in osteogenic differentiation. (A–B) Cell cycle profiles of hBMSCs from hCTR and hB-ALL patients analyzed by FCA. (C) Proliferation of hBMSCs from hCTR and hB-ALL patients evaluated by CCK-8 assay. (D) ALP staining after 7 days of osteogenic induction. (E) ARS staining and calcium nodule quantification after 21 days of osteogenic induction. (F–G) Western blot and quantification of osteogenic proteins (COL1A1, ALP, RUNX2). (H) qRT-PCR analysis of osteogenic genes (*OCN*, *RUNX2*, *ALP*, *COL1A1*, *BMP2*, *OPN*) (n = 3; ∗*P* < 0.05, ∗∗*P* < 0.01, ∗∗∗*P* < 0.001, ∗∗∗∗*P* < 0.0001).

After confirming the proliferative and differentiation potential of hBMSCs, we compared BMSCs derived from hB-ALL patients and hCTR individuals. Cell cycle analysis showed that hB-ALL BMSCs were predominantly arrested in the S phase, with reduced G_2_/M phase populations (Fig. 2A and B). CCK-8 assays revealed significantly decreased proliferation in hB-ALL BMSCs relative to hCTR (Fig. 2C). These results collectively indicate that the proliferative capacity of hBMSCs is markedly reduced in B-ALL.

Osteogenic differentiation assays demonstrated weakened osteogenic potential in B-ALL hBMSCs, reflected by reduced ALP activity (Fig. 2D) and a 1.67-fold decline in ARS-stained calcium nodule formation after 21 days of induction (Fig. 2E). Consistent to these findings, qRT-PCR and western blot analysis showed a significant downregulation of osteogenic markers, including *OCN* (5.66-fold decrease), *RUNX2* (14.38-fold decrease), *ALP* (2.33-fold decrease), *COL1A1* (5.65-fold decrease), *BMP2* (7.82-fold decrease), and *OPN* (9.01-fold decrease), along with reduced protein expression of COL1A1, ALP, and RUNX2 (Fig. 2F–H).

Conversely, adipogenic differentiation was enhanced in B-ALL hBMSCs, as evidenced by increased lipid droplet accumulation (Supplementary Fig. 3A) and elevated expression of the adipogenic marker ADIPOQ (Supplementary Fig. 3B). In contrast, chondrogenic differentiation was impaired, with hB-ALL BMSCs showing decreased expression of SOX9 protein (Supplementary Fig. 3C) and downregulated chondrogenic genes (*SOX9*, *AGC1*, *MMP13*, *COL2A1*) (Supplementary Fig. 3D), accompanied by reduced proteoglycan synthesis (Supplementary Fig. 3E). Collectively, compared with age-matched healthy hBMSCs, B-ALL hBMSCs exhibit reduced proliferation, osteogenesis, and chondrogenesis, alongside enhanced adipogenic differentiation potential.

### 3.4 Multi-omics analysis identifies SFRP1 as a key driver of BMSC dysfunction in the pediatric B-ALL patients

Proteomic sequencing revealed 118 differential proteins (DEPs) between hB-ALL and hCTR BMSCs, including 56 upregulated and 62 downregulated in B-ALL hBMSCs (Supplementary Fig. 4A and B). KEGG pathway analysis further indicated suppressed apoptotic pathways and elevated expression of fatty acid metabolism-related proteins (Supplementary Fig. 4C). GO enrichment analysis showed that these DEPs were primarily involved in bone remodeling and DNA replication, with downregulation of key proteins linked to osteogenesis, chondrogenesis, and cell cycle regulation, including two central BMP signaling regulators (Supplementary Fig. 4D).

**Fig. 3:**
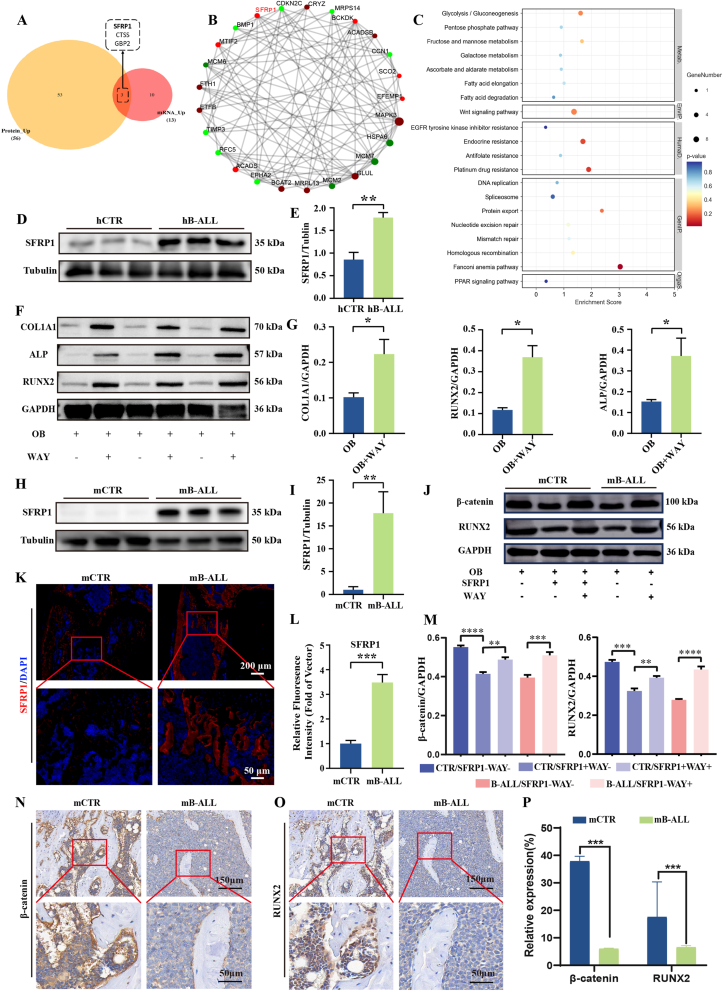
SFRP1 suppresses osteogenesis of B-ALL–derived BMSCs through the Wnt/β-catenin pathway. (A) Venn diagram showing overlap between upregulated mRNA and protein datasets. (B) Protein-protein interaction network of DEPs (red: upregulated; green: downregulated). (C) KEGG pathway enrichment analysis of DEGs. (D, E) Quantification of SFRP1 protein expression in hBMSCs from hCTR and hB-ALL patients. (F, G) Quantification of osteogenic proteins (COL1A1, ALP, RUNX2) in hBMSCs from hB-ALL with WAY-316606. (H, I) Quantification of SFRP1 protein expression in mBMSCs from mCTR and mB-ALL mice. (J, M) β-catenin and RUNX2 protein expression in mBMSCs with different treatments. (K, L) Immunofluorescence imaging and quantification of SFRP1 (red) in mouse trabecular bone. (N–P) Immunohistochemical analysis of β-catenin and RUNX2 expression in mouse trabecular bone (n = 3; ∗*P* < 0.05, ∗∗*P* < 0.01, ∗∗∗*P* < 0.001, ∗∗∗∗*P* < 0.0001).

To expand upon these findings, transcriptomic analysis of hB-ALL and hCTR BMSCs was performed. RNA-seq identified 645 differentially expressed genes (DEGs), comprising 259 upregulated and 386 downregulated transcripts (Supplementary Fig. 4E and F). Integrated multi-omics analysis revealed three overlapping targets (*SFRP1*, *CTSS*, and *GBP2*) that were consistently upregulated at both mRNA and protein levels. Among these, *SFRP1* exhibited the highest combined expression, identifying it as a principal regulatory candidate (Fig. 3A). Protein-protein interaction (PPI) network analysis linked *SFRP1* to osteogenic and skeletal development pathways involving *CCN1*, *MAPK3*, *MCM7*, *MCM2*, *EPHA2*, and *BMP1* (Fig. 3B). KEGG pathway mapping indicated significant modulation of the Wnt signaling, PPAR signaling, and fatty acid metabolism pathways (Fig. 3C). Gene set enrichment analysis (GSEA) further demonstrated that Wnt signaling and bone remodeling signatures were downregulated in B-ALL (Supplementary Fig. 4G–I). Together, these results indicate that *SFRP1* serves as a critical driver of BMSC dysfunction in B-ALL and may contribute to leukemia-associated bone loss.

### 3.5 SFRP1 inhibits osteogenic differentiation in B-ALL-derived hBMSCs

To validate these findings, protein and RNA were extracted from both hCTR and B-ALL-derived hBMSCs to assess SFRP1 expression. Western blot (WB) and qRT-PCR analyses revealed that SFRP1 was significantly upregulated at both protein and mRNA levels in hBMSCs from B-ALL patients compared to hCTR (Fig. 3D and E; Supplementary Fig. 5A), consistent with the integrated multi-omics results.

To examine the functional impact of SFRP1 on osteogenic and adipogenic differentiation in hB-ALL BMSCs, *in vitro* studies were performed employing the SFRP1 inhibitor WAY-316606. Pharmacological inhibition of SFRP1 markedly restored osteogenic capacity, as shown by increased expression of key osteogenic markers (COL1A1, ALP, RUNX2) (Fig. 3F and G) and enhanced alizarin red S staining, indicating greater mineralized matrix deposition in OB + WAY cultures compared with OB-only groups (Supplementary Fig. 5B). Conversely, WAY-316606 treatment suppressed adipogenic differentiation, demonstrated by reduced lipid droplet formation (Oil Red O staining; Supplementary Fig. 5C) in Adipocyte + WAY versus Adipocyte groups.

To further corroborate these findings, BMSCs were isolated from murine B-ALL (mB-ALL) to evaluate SFRP1 expression. WB analysis showed significantly elevated SFRP1 expression in mBMSCs from mB-ALL mice compared with mCTR controls (Fig. 3H and I). This SFRP1 overexpression in mB-ALL BMSCs mirrored the pathological profile seen in patient-derived cells, confirming the translational relevance of the murine model. Inhibition of SFRP1 in mBMSCs also elevated β-catenin expression and increased expression of key osteogenic markers RUNX2 (Fig. 3J–M).

Bone tissues from mB-ALL mice were also analyzed for SFRP1 expression. Immunofluorescence staining revealed increased SFRP1 levels in trabecular bone beneath the growth plate in mB-ALL mice relative to mCTR (Fig. 3K and L). Immunohistochemical analysis further demonstrated that β-catenin expression in osteoblast cytoplasm along trabecular surfaces was reduced in mB-ALL mice (Fig. 3N–P), accompanied by decreased nuclear translocation of β-catenin (Fig. 3N) and reduced nuclear expression of RUNX2 (Fig. 3O and P). Collectively, these results indicate that SFRP1 functions as a major driver of BMSC dysfunction in B-ALL by modulating the Wnt/β-catenin signaling pathway.

### 3.6 Characterization of shSFRP1@Lipo-E7

A critical consideration for developing the *SFRP1* silencing system was minimizing its interaction with leukemia cells *in vivo*, given *SFRP1*'s established role as a tumor suppressor regulating proliferation, migration, and drug resistance. To achieve targeted silencing of *SFRP1* in B-ALL BMSCs, a lentiviral delivery platform (shSFRP1@Lipo-E7) was engineered by encapsulating shSFRP1-carrying lentiviruses within E7 peptide-modified liposomes.

**Fig. 4:**
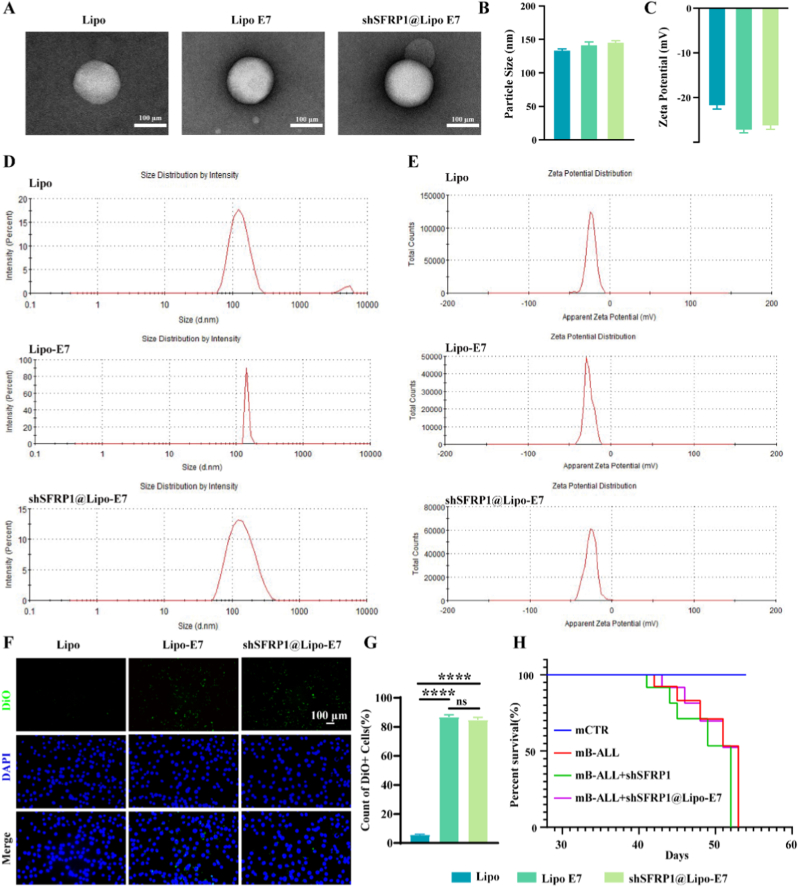
Characterization and uptake capacity of liposomes. (A) Representative TEM images of various liposomes. (B, D) Particle size of Lipo, Lipo-E7, shSFRP1@Lipo-E7. (C, E) Zeta potential of Lipo, Lipo-E7, shSFRP1@Lipo-E7. (F, G). Assessment of differential cellular uptake of various liposomes by mBMSCs. (H). Evaluation of the effects of various treatment modalities on the survival duration in B-ALL mice. ∗∗∗∗*P* < 0.0001, ns: no significance.

Transmission electron microscopy (TEM) confirmed spherical liposome morphology, with E7 functionalization and lentiviral encapsulation maintaining structural integrity (Fig. 4A). Dynamic light scattering analysis showed uniform hydrodynamic diameters: Lipo (135.1 ± 2.55 nm), Lipo-E7 (139.53 ± 5.08 nm), and shSFRP1@Lipo-E7 (145 ± 4.05 nm), along with stable polydispersity indices (Fig. 4B–D). Zeta potential measurements indicated a shift from −22.1 ± 0.78 mV (Lipo) to −27.9 ± 0.65 mV (Lipo-E7) and −26.4 ± 0.68 mV (shSFRP1@Lipo-E7), confirming successful surface modification (Fig. 4C–E). DiO fluorescence assays demonstrated enhanced uptake of E7-functionalized liposomes (Lipo-E7, shSFRP1@Lipo-E7) by mBMSCs compared with unmodified Lipo, with lentiviral payloads not affecting uptake efficiency (Fig. 4F and G).

The *in vivo* biocompatibility of the delivery system was rigorously evaluated. Flow cytometry analysis showed comparable human CD45^+^ cell proportions in peripheral blood between hB-ALL + PBS and hB-ALL + shSFRP1@Lipo-E7 groups (Supplementary Fig. 6), suggesting no off-target effects on leukemic proliferation. Survival analysis further confirmed biosafety, with no mortality differences between treated and untreated hB-ALL cohorts (Fig. 4H).

H&E staining of major organs (heart, kidneys, brain, spleen, lungs, liver) from B-ALL mice treated with shSFRP1@Lipo-E7 for 3 weeks revealed no histopathological abnormalities in any experimental groups, including PBS, Lipo, Lipo-E7, NC@Lipo-E7, shSFRP1, NC, and shSFRP1@Lipo-E7 cohorts (Supplementary Fig. 7). These results collectively confirm the structural stability and biosafety of the liposomal delivery system.

### 3.7 shSFRP1@Lipo-E7 intervention mitigates bone loss and restores bone remodeling balance in B-ALL mice

As shown *in vitro*, blocking the SFRP1 pathway effectively enhances the osteogenic differentiation of hBMSCs. To determine whether targeted silencing of *SFRP1* could also alleviate bone loss *in vivo*, different liposomal formulations or PBS were administered to B-ALL mice, followed by structural and biochemical assessments.

**Fig. 5:**
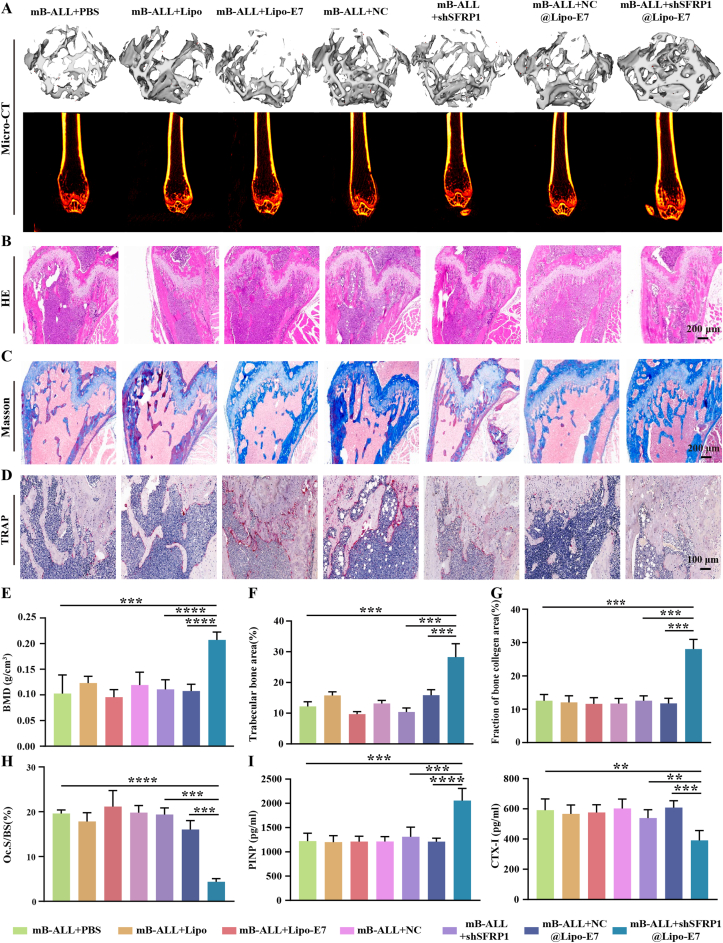
shSFRP1@Lipo-E7 rescues bone loss in B-ALL mice. (A, E) Micro-CT reconstruction of femoral cancellous bone, showing 3D (top) and sagittal 2D (bottom) views with quantitative analysis of BMD. (B, F) H&E staining of proximal femurs and quantification of trabecular bone area. (C, G) Masson's trichrome staining of proximal femurs and quantification of collagen volume fraction. (D, H) TRAP staining of proximal femurs and quantification of osteoclast surface area per bone surface (Oc.S/BS). (I) PINP and CTX-I levels in peripheral blood of B-ALL mice across treatment groups (n = 5; ∗∗*P* < 0.01, ∗∗∗*P* < 0.001, ∗∗∗∗*P* < 0.0001).

Micro-CT analysis revealed markedly improved trabecular organization, reduced cancellous bone loss, and decreased marrow space expansion in mB-ALL + shSFRP1@Lipo-E7 mice compared to mB-ALL + PBS, mB-ALL + NC@Lipo-E7, and mB-ALL + shSFRP1 groups (Fig. 5A). No significant bone restoration was observed in groups treated with free shSFRP1 or control liposomes (Lipo, LV-NC, Lipo-E7). Quantitative analysis confirmed significant therapeutic efficacy, evidenced by increased BMD (0.207 ± 0.016 g/cm^3^ vs 0.105 ± 0.018 g/cm^3^, P < 0.0001), BV/TV (28.63 ± 2.60% vs 12.59 ± 3.17%, P < 0.0001), Tb.Th (0.117 ± 0.014 mm vs 0.085 ± 0.007 mm, P < 0.001), and Tb.N (2.44 ± 0.20 mm^−1^ vs 1.47 ± 0.31 mm^−1^, P < 0.001), alongside decreased SMI (1.879 ± 0.159 vs 2.366 ± 0.149, P < 0.001) and Tb.Sp (0.287 ± 0.013 mm vs 0.361 ± 0.050 mm, P < 0.05) in the shSFRP1@Lipo-E7 group (Fig. 5E; Supplementary Fig. 8D).

Histological evaluation further supported these findings. H&E staining revealed increased trabecular density, improved cortical-trabecular connectivity, and enhanced bone structural integrity in distal femurs of shSFRP1@Lipo-E7-treated mice compared to PBS and shSFRP1 groups (Fig. 5B–F; Supplementary Fig. 8A). Masson's trichrome staining showed increased collagen deposition (intense blue regions) in treated mice, indicative of active bone matrix synthesis (Fig. 5C–G; Supplementary Fig. 8B). TRAP staining demonstrated markedly reduced osteoclast activity in the shSFRP1@Lipo-E7 group relative to controls (Fig. 5D–H; Supplementary Fig. 8C). Together, these results confirm that shSFRP1@Lipo-E7 effectively mitigates bone loss in B-ALL mice by rebalancing bone remodeling dynamics.

Serum biomarker analysis revealed significantly elevated PINP levels (2058 ± 250.3 pg/mL) and decreased CTX-I levels (391.2 ± 65.4 pg/mL) in shSFRP1@Lipo-E7-treated mice compared with PBS and shSFRP1 groups (Fig. 5I). No significant biomarker changes were observed with shSFRP1 alone or control formulations (Lipo, LV-NC, Lipo-E7, NC@Lipo-E7, Fig. 5I).

These findings collectively demonstrate that targeted *SFRP1* silencing through E7-functionalized liposomes restores the osteogenic-osteoclastic balance, promoting bone formation while suppressing pathological resorption in the leukemic bone microenvironment. Collectively, *SFRP1* emerges as a promising therapeutic target for preventing leukemia-associated bone loss.

### 3.8 shSFRP1@Lipo-E7 facilitates β-catenin expression and nuclear translocation

Nuclear translocation of β-catenin is a hallmark of Wnt/β-catenin pathway activation and a direct indicator of its biological activity, particularly in regulating osteogenic differentiation. Therefore, the level of nuclear β-catenin serves as a reliable marker of pathway activation [32].

**Fig. 6:**
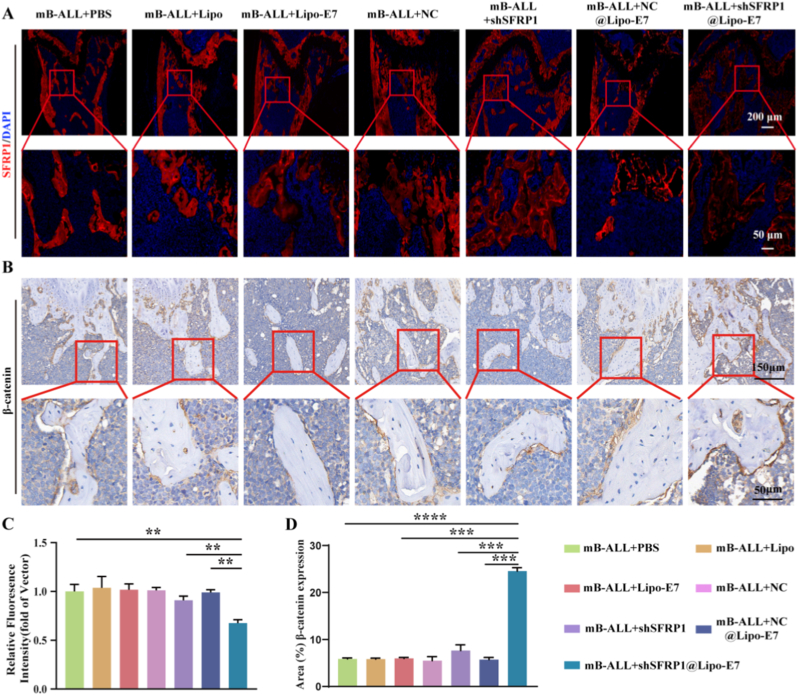
SFRP1 inhibition enhances β-catenin expression and nuclear translocation (A, C) Immunofluorescence analysis and quantification of SFRP1 expression (red) in femoral sections of mB-ALL mice across treatment groups. (B, D) β-catenin expression and nuclear localization in femoral trabeculae across treatment groups (n = 5; ∗∗*P* < 0.01, ∗∗∗*P* < 0.001, ∗∗∗∗*P* < 0.0001).

To determine whether shSFRP1@Lipo-E7 exerts bone-targeted regulation of the Wnt/β-catenin pathway via *SFRP1* suppression, immunofluorescence and immunohistochemical analyses were performed. Immunofluorescence staining of femoral trabecular bone revealed significantly decreased *SFRP1* expression in the mB-ALL + shSFRP1@Lipo-E7 group (0.676 ± 0.035) compared to the mB-ALL + PBS group (1 ± 0.072), while the mB-ALL + shSFRP1 group (0.91 ± 0.043) showed no substantial reduction (Fig. 6A–C). These results highlight the superior knockdown efficiency achieved by E7-functionalized liposomal delivery compared to free shSFRP1.

**Fig. 7:**
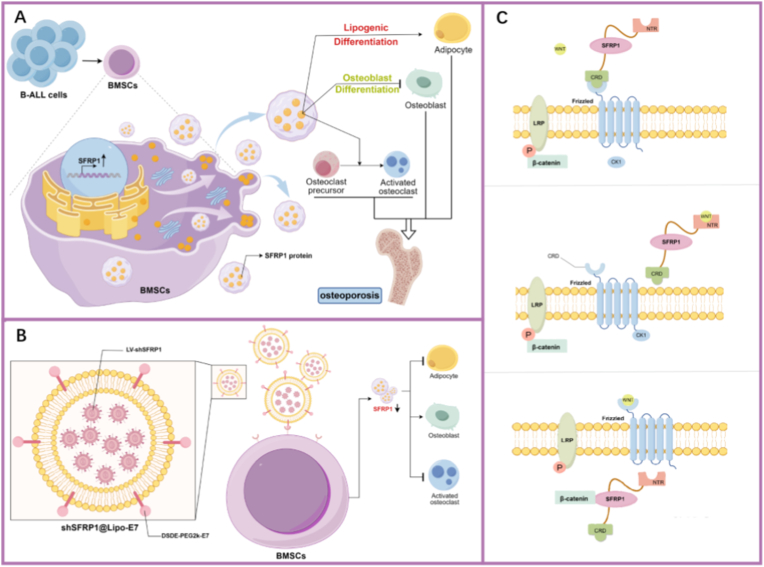
shSFRP1@Lipo-E7 targets BMSCs to regulate the SFRP1/β-catenin signaling pathway, promoting osteogenic differentiation of BMSCs and alleviating bone loss induced by B-ALL. (A) B-ALL cells induce abnormal elevation of SFRP1 in BMSCs, promoting adipogenic differentiation and disrupting the osteogenic-osteoclastic coupling balance, ultimately leading to bone loss. (B) Tail vein delivery of shSFRP1-loaded BMSC-targeted liposomes downregulates SFRP1 to enhance BMSC osteogenic differentiation, suppress adipogenic differentiation, and indirectly inhibit osteoclastogenesis by remodeling the bone microenvironment. (C) SFRP1 inhibit Wnt signaling by promoting the phosphorylation of β-catenin and reducing its nuclear translocation.

Furthermore, β-catenin expression was markedly elevated in shSFRP1@Lipo-E7 (24.554 ± 0.721) compared to mB-ALL + PBS controls (5.897 ± 0.2), accompanied by enhanced nuclear translocation within trabecular osteocytes (Fig. 6B–D). These observations verify that targeted *SFRP1* silencing through shSFRP1@Lipo-E7 reactivates Wnt/β-catenin signaling, thereby promoting osteogenesis and mitigating leukemia-associated bone deterioration. In sum, we confirm that B-ALL cells induce abnormal elevation of SFRP1 in BMSCs, which is a key factor in B-ALL-induced bone loss. Furthermore, shSFRP1@Lipo-E7-mediated targeted regulation of BMSCs restores osteogenic activity, reverses trabecular bone loss, and normalizes serum bone remodeling biomarkers (Fig. 7).

## 4 Discussion

The pathogenesis of bone loss in pediatric B-ALL represents a multifactorial process involving leukemic infiltration, microenvironmental remodeling, and dysregulated bone metabolism. Although chemotherapy-induced skeletal toxicity is well documented, emerging evidence indicates that leukemia itself acts as a primary driver of osteopenia, independent of treatment effects [2]. The present study revealed that 33.8% of adolescent B-ALL patients exhibited significant vertebral bone loss at diagnosis, consistent with prior findings showing that leukemic infiltration disrupts bone homeostasis through direct cellular invasion and paracrine mechanisms [6]. Leukemic cells physically occupy the marrow cavity, compress vascular channels, and increase intramedullary pressure, thereby impairing osteoblast-mediated bone formation [33]. Simultaneously, leukemia-derived cytokines such as RANKL, IL-6, and CCL3 alter the RANKL/OPG balance, promoting excessive osteoclast activation and bone resorption [6]. These pathophysiological features are faithfully reproduced in murine B-ALL models, which display trabecular thinning, decreased bone mineral density, and heightened osteoclast activity, mirroring human skeletal pathology. Furthermore, 12.2% of patients presented vertebral CT values below the −2 SD threshold of healthy controls, meeting the diagnostic criteria for osteoporosis. The age-dependent vulnerability of adolescents likely reflects puberty-associated hormonal fluctuations and accelerated bone turnover, rendering the developing skeleton particularly susceptible to leukemic insults [34].

The core of B-ALL–associated bone loss lies in BMSC dysfunction, as these cells serve as osteoblast progenitors essential for skeletal maintenance. Our data reveal two principal abnormalities in B-ALL–derived hBMSCs. First, these cells exhibit decreased proliferation and S-phase arrest, leading to reduced cellular availability. Second, they demonstrate enhanced adipogenic differentiation accompanied by impaired osteogenic differentiation and decreased mineralization capacity. Additionally, chondrogenic differentiation potential is also suppressed, indicating possible disruption of endochondral ossification. Collectively, these results suggest that the lineage imbalance of BMSCs—specifically, the shift away from osteogenic and chondrogenic lineages toward adipogenesis—represents a major mechanism contributing to skeletal deterioration in pediatric B-ALL.

Multi-omics profiling identified SFRP1 as a key mediator of BMSC impairment, with patient-derived cells showing significant upregulation of SFRP1 at both mRNA and protein levels. Inhibition of SFRP1 function in hBMSCs enhanced osteogenesis while suppressing adipogenesis, confirming its central regulatory role in leukemic bone pathology. Mechanistically, SFRP1 suppresses Wnt/β-catenin signaling by binding to Wnt ligands or Frizzled receptors and directly binding to the cytoplasmic β-catenin, preventing β-catenin nuclear translocation and thereby blocking osteoblast differentiation [32]. It is important to emphasize that SFRP1-mediated regulation of Wnt/β-catenin exhibits significant cell type dependency. In BMSCs, SFRP1 upregulation primarily exacerbates bone loss; however, this change does not equate to the Wnt pathway status within leukemic cells. Therefore, assessing the pathological significance of the SFRP1-Wnt axis requires distinguishing between donor and recipient cells, and cannot be simply extrapolated as an overall effect. In B-ALL, sustained SFRP1 overexpression not only suppresses osteoblastogenesis but also facilitates osteoclastogenesis, as reflected by elevated osteoclast surface-to-bone surface ratios. Unlike AML, where *SFRP1* is epigenetically silenced [12], our study demonstrates SFRP1 upregulation in B-ALL, highlighting lineage-specific regulatory divergence. Previous studies have demonstrated that AML establishes an osteolytic leukemic-supportive microenvironment through DKK1-mediated Wnt inhibition and IL-6/JAK2/STAT3-driven BMSC senescence [12,35]. In contrast, our findings suggest that B-ALL pathogenesis relies more heavily on SFRP1-driven osteoblast suppression and osteoclast activation. Elevated SFRP1 expression in B-ALL BMSCs may also represent a feedback mechanism to counteract excessive Wnt activity in the leukemic niche, which otherwise sustains leukemia stem cell quiescence and chemoresistance [36]. This discrepancy suggests that leukemia-associated bone disease may be jointly shaped by differences in the secretory profiles of leukemic cells, the composition of BMSC subpopulations, and receptor expression. Under disease conditions, distinct subtypes may selectively rely on different antagonistic molecules (such as SFRP1 or DKK1) to regulate Wnt-mediated bone metabolism. This underscores the necessity of precisely targeting and silencing SFRP1 in BMSCs to avoid systemic Wnt disruption—thereby restoring osteogenic function without exacerbating leukemia progression.

Despite the mechanistic insights into leukemia-induced bone loss, current therapeutic options remain inadequate. Existing interventions, including bisphosphonates and denosumab, provide limited efficacy and carry significant risks of hypocalcemia, nephrotoxicity, and atypical fractures [19,37]. Moreover, their lack of cellular specificity and potential to promote tumor proliferation limit clinical applicability. These shortcomings highlight the necessity for niche-directed therapies that directly address the underlying BMSC dysfunction rather than targeting osteoblasts or osteoclasts in isolation. The development of the shSFRP1@Lipo-E7 nanoplatform represents a significant advancement in this regard. By encapsulating lentiviral shRNA within E7 peptide-functionalized liposomes, selective *SFRP1* silencing in BMSCs was achieved, minimizing systemic toxicity and off-target effects. The E7 peptide binds CD71, a transferrin receptor abundantly expressed on BMSCs, enabling precise cellular targeting, while the lipid nanoparticle structure enhances circulatory stability and prolongs therapeutic efficacy [23]. Preclinical studies confirmed that shSFRP1@Lipo-E7 restored osteogenic activity, reversed trabecular bone loss, and normalized serum biomarkers of bone remodeling (Fig. 7). These outcomes align with previous studies showing that Wnt pathway activation in BMSCs promotes osteogenesis while inhibiting adipogenesis [38], as well as research demonstrating RNA interference-based modulation of the bone marrow niche in other malignancies [39].

Nonetheless, certain limitations must be addressed before clinical translation. The underlying mechanisms by which B-ALL cells induce SFRP1 secretion in BMSCs warrant further investigation. Current research indicates that Th17 cells and IL-17A expression are significantly elevated in patients with B-cell acute lymphoblastic leukemia (B-ALL). IL-17A promotes B-ALL progression [40]. Other studies suggest that IL-17A inhibits the osteogenic differentiation of bone marrow mesenchymal stem cells (BMSCs) via the Wnt signaling pathway. IL-17A induces upregulation of sFRP1 expression in BMSCs while downregulating Wnt3 and Wnt6 expression. Conversely, sFRP1-shRNA eliminates IL-17A's inhibitory effect on osteogenic differentiation in BMSCs and induces upregulation of Wnt3 and Wnt6 expression in the Wnt signaling pathway [41]. Therefore, we speculate elevated Th17 cells in pediatric B-ALL patients may secrete increased IL-17A, leading to heightened SFRP1 expression in MSCs. The single-center cohort restricts generalizability, necessitating multi-center validation across diverse patient populations. Furthermore, although shSFRP1@Lipo-E7 exhibited excellent short-term biosafety *in vivo*, long-term risks related to lentiviral vector integration, such as insertional mutagenesis, remain unresolved. Future investigations should consider non-integrating mRNA or CRISPR-based epigenetic editing approaches to achieve stable yet safer gene silencing [42]. Co-culture studies with osteoclast precursors could also clarify whether SFRP1 regulates RANKL expression directly or indirectly via cytokine signaling. From a translational perspective, large-scale production of lipid nanoparticles under Good Manufacturing Practice (GMP) conditions must be optimized to support clinical application [43]. Additionally, combinatorial strategies—such as co-delivering SFRP1 inhibitors alongside immunotherapies—may enhance anti-leukemic efficacy while preserving bone integrity. For example, PD-1/PD-L1 blockade has been shown to potentiate CAR-T cell activity against B-ALL [44]; integrating such immunotherapies with niche-targeted treatments could provide synergistic benefits. Addressing these scientific, technical, and regulatory challenges will be essential for advancing microenvironment-targeted therapies from bench to bedside.

In conclusion, this study establishes SFRP1 as a pivotal mediator of BMSC dysfunction and bone loss in pediatric B-ALL. Through a BMSC-targeted nanoplatform, we achieved selective modulation of Wnt signaling, effectively restoring osteogenesis and mitigating leukemia-induced skeletal damage without exacerbating disease progression. These findings not only provide mechanistic insights into the leukemia-bone marrow niche interaction but also highlight the therapeutic promise of microenvironment-directed strategies in hematologic malignancies. The proposed platform offers a viable approach for precision modulation of stromal cell dysfunction, paving the way for adjunctive treatments that complement conventional anti-leukemic therapies and enhance patient quality of life.

## Author contributions

Mengxia Li: Writing - original draft, Visualization, Validation, Software, Methodology, Investigation, Data curation, Project administration. Xu He: Writing - original draft, Formal analysis, Methodology, Conceptualization. Xingzhi Liu: Writing - review & editing, Writing - original draft, Investigation, Conceptualization. Mimi Chen: Validation. Qian Sun: Methodology, Software. Ronghui Yu: Investigation, Data curation. Wendong Liu: Software. Qi Wang: Project administration. Guanghao Su:Investigation, Supervision, Resources. Qin Shi: Writing - review & editing, Writing - original draft, Supervision, Funding acquisition. Xiaodong Wang:Writing - review & editing, Funding acquisition, Formal analysis, Resources.

## Ethics statement

This retrospective clinical study was approved by the Medical Ethics Committee of Children's Hospital of Soochow University (No. 2024CS015) and conducted in accordance with the Declaration of Helsinki. All patient data were anonymized to ensure complete privacy protection. Bone marrow (BM) specimens used for hBMSC extraction were obtained from leftover samples following diagnostic testing in the hospital's Laboratory Department, without causing additional discomfort to the children.

For animal experiments, 6-week-old female NOD-SCID gamma (NSG) mice were purchased from the Shanghai Model Organisms Center and housed under specific pathogen-free (SPF) conditions at Soochow University. All animal procedures were approved by the Experimental Animal Ethics Committee of Soochow University (No. SUDA20230625A02).

## Declaration of generative AI in scientific writing

The authors declare that they have not used AI to write or edit this manuscript.

## Funding statement

This work was supported by grants from the 10.13039/501100001809National Natural Science Foundation of China (82372457), the 10.13039/501100012246Priority Academic Program Development of Jiangsu Higher Education Institutions (10.13039/501100012246PAPD), Project of 10.13039/501100002949Jiangsu Province Key Research and Development Plan (BK20251792,BE2022732), 10.13039/100016086Key Laboratory in Science and Technology Development Project of Suzhou (CN, SZS2024012), 10.13039/501100007824Soochow University 2024 Doctoral Research Start-up Fund (RC202416), Soochow Medical College Clinical Educator (undergraduate education oriented) (MA13100123), Shanghai Huangpu District Health Commission (HLQ202501, 2025QN02).

## Declaration of competing interest

The authors declare no conflict of interest relevant to this article.

## Data Availability

The datasets used in this study are available from the corresponding author upon reasonable request.
