# Supplementary material for: Silencing SFRP1 in bone mesenchymal stem cells alleviates pediatric B-ALL-driven bone loss by activating Wnt/β-catenin signaling
Source: J Orthop Translat. 2026 Mar 24;57:101071. doi: 10.1016/j.jot.2026.101071 (PMC13049614; doi:10.1016/j.jot.2026.101071)
Supplement: Multimedia component 1 [file mmc1.docx]

**Supporting Information**

**
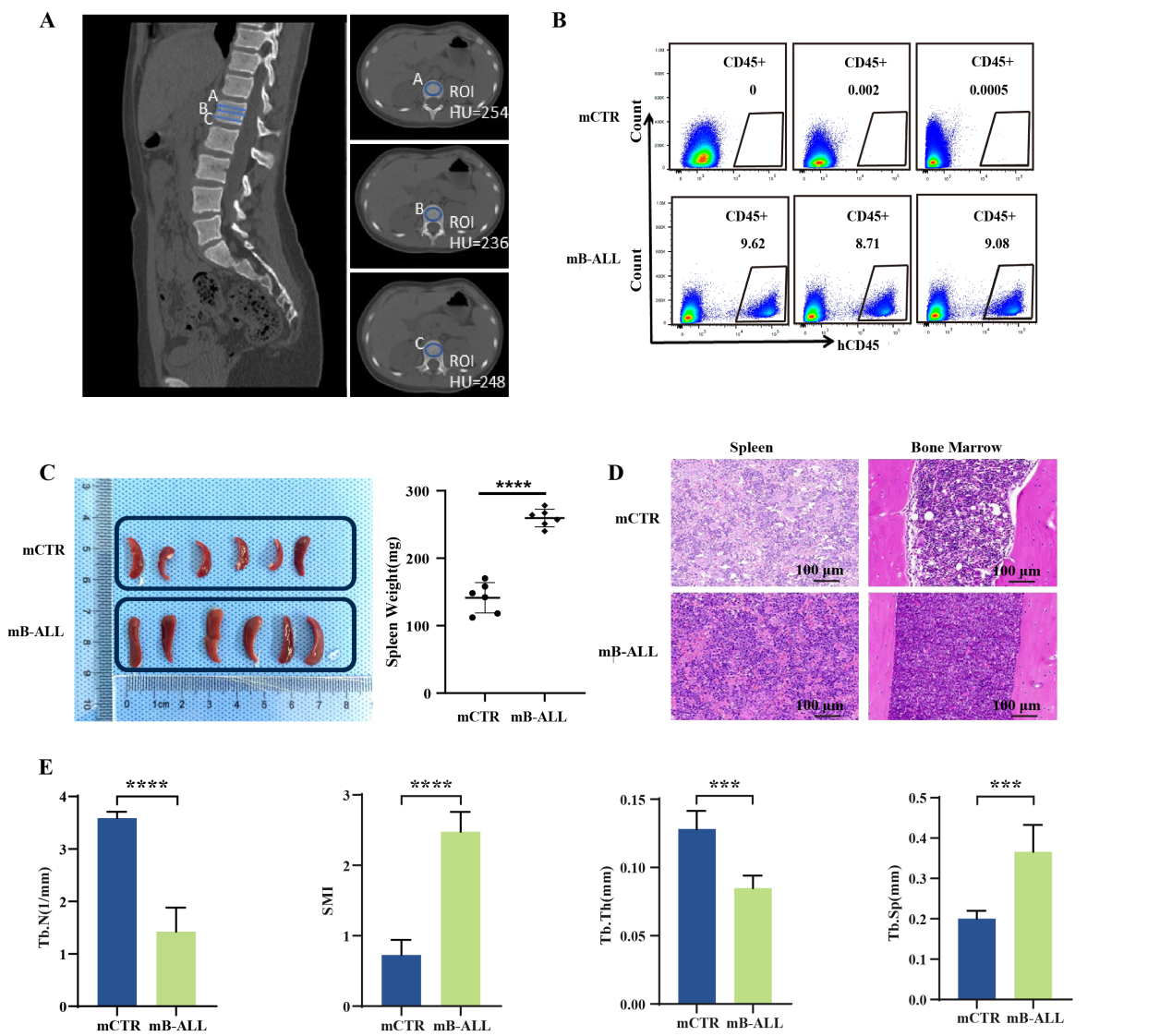
**

**Supplementary Figure 1**. Vertebral CT value measurement in pediatric B-ALL patients and establishment of B-ALL mouse models. **(A) Schematic illustration of ROI selection for vertebral HU value measurements of pediatric B-ALL patients. A: upper subterminal plate; B: vertebral middle level; C: lower subterminal plate. (B)** The population of CD45^+^ hB-ALL1 cells in the peripheral blood of mice detected by FCA (n=3). (C) The gross observation and weight of the spleens of the mB-ALL and mCTR mice after 28 d of modeling. (D) Leukemic infiltration in spleen and bone marrow by H&E staining (scale bars: 100 μm). (E) The bone parameters of the femurs scanned by micro-CT. (n=6, ******P* <0.001, *****P* <0.0001)**

**
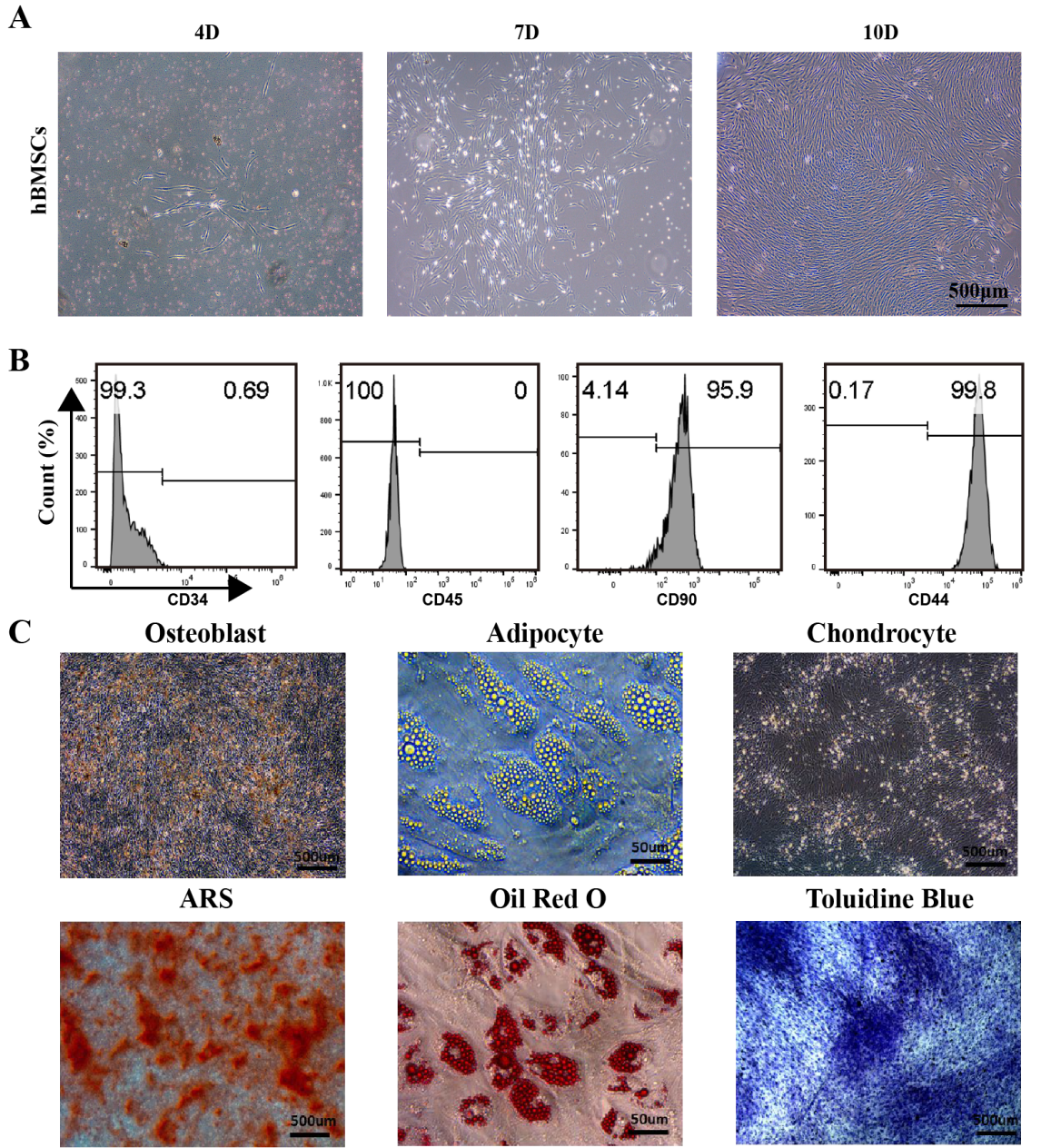
**

**Supplementary Figure 2**. Characterization and trilineage differentiation of human BMSCs. (A) Morphology of primary hBMSCs from healthy donors under light microscopy. (B) Flow cytometric analysis of surface markers of hBMSCs. (C) Trilineage differentiation of hBMSCs: representative ARS, Oil Red O, and toluidine blue staining images.


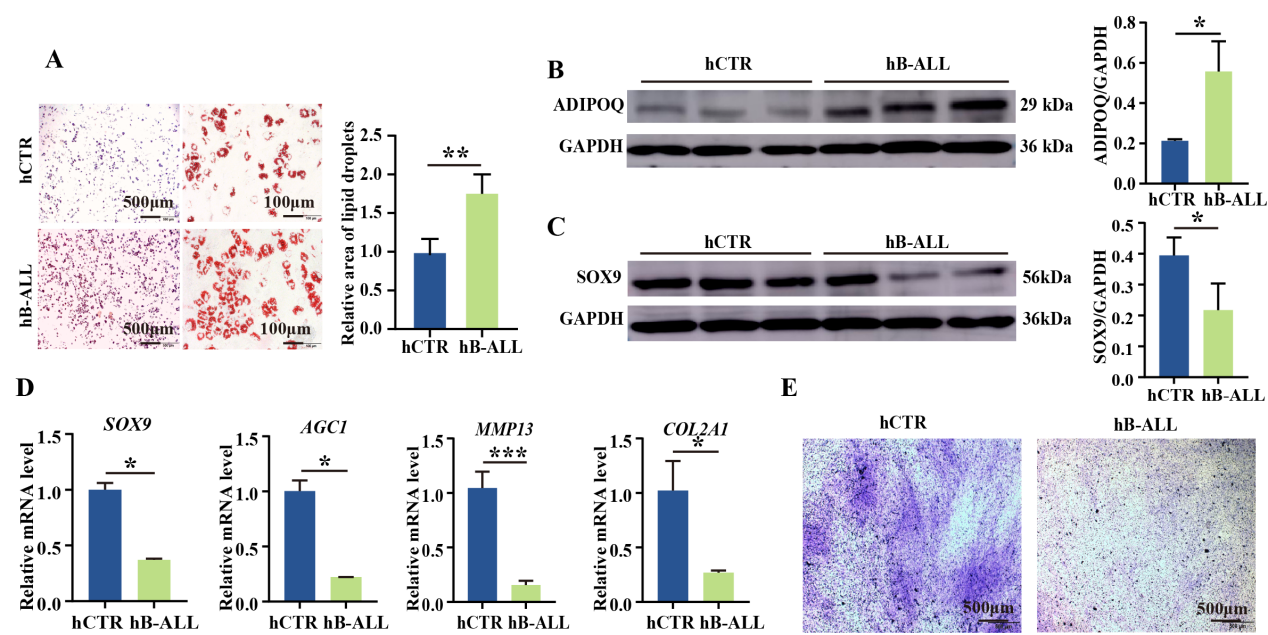


**Supplementary Figure 3**. Evaluation of differentiation capacity of different hBMSCs from B-ALL patients. (A) Representative Oil Red O staining of hBMSCs from hCTR and hB-ALL patients after 21 days of adipogenic induction. (B) Expression of adipogenic marker protein ADIPOQ in hBMSCs from hCTR and hB-ALL patients. (C) Expression of chondrogenic marker protein SOX9 in hBMSCs from hCTR and hB-ALL patients. (D) Expression of chondrogenic genes *SOX9*, *AGC1*, *MMP13*, and *COL2A1* in hBMSCs from hCTR and hB-ALL patients. (E) Representative toluidine blue staining of hBMSCs from hCTR and hB-ALL patients after 14 days of chondrogenic induction. n=3; ****P* <0.05,** ******P* <0.001.**


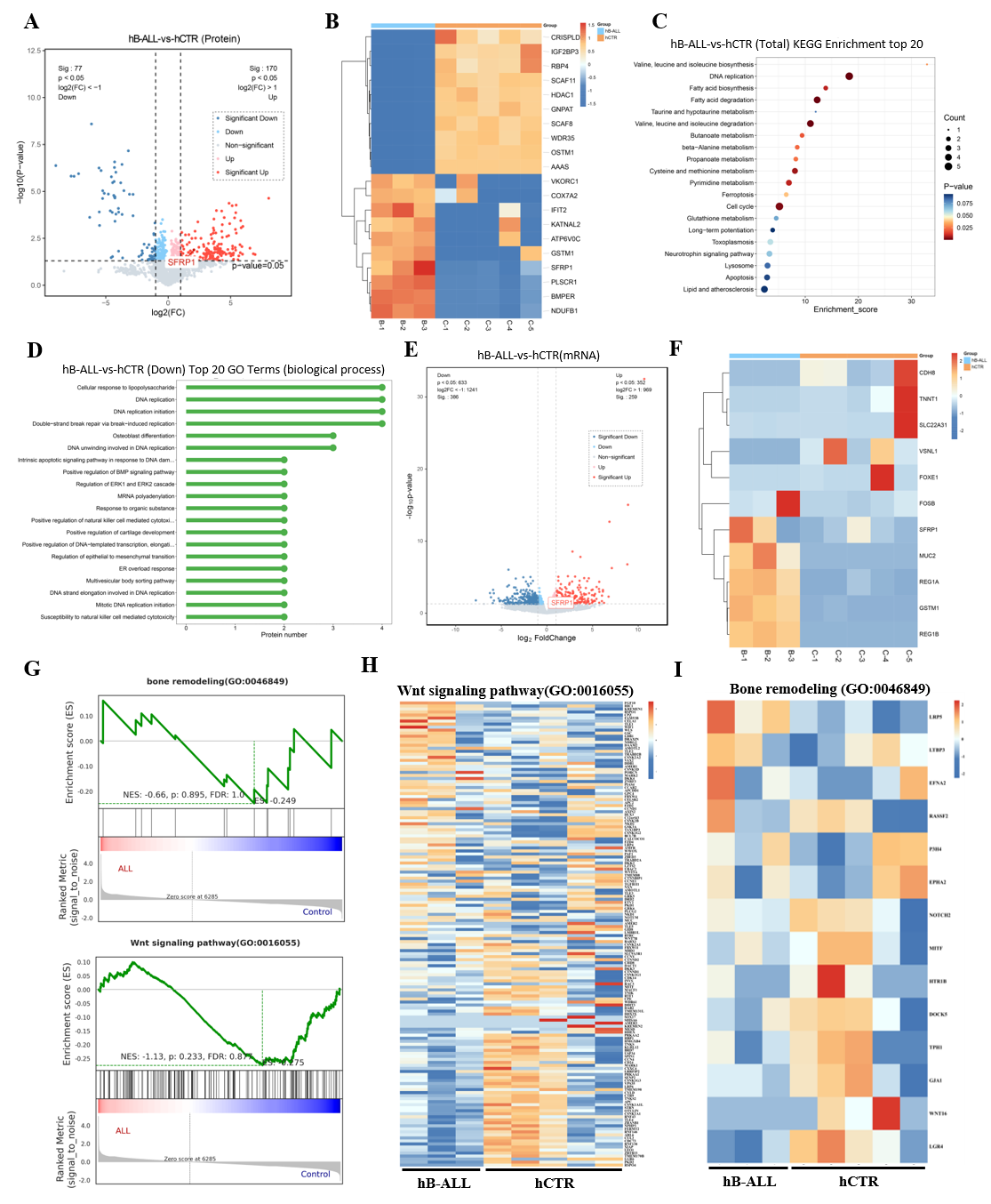


**Supplementary Figure 4**. Multi-omics analysis of human BMSCs derived from hCTR and hB-ALL. (A) Volcano plot of DEPs (red: upregulated; blue: downregulated). (B) Expression heatmap of DEPs between groups. (C) Top 20 KEGG Enrichment of total DEPs. (D) Top 20 GO terms enriched among downregulated proteins. (E) Volcano plot of DEGs (red: upregulated; blue: downregulated). (F) Expression heatmap of DEGs between groups. (G) GSEA showing enrichment of DEGs in bone remodeling and Wnt signaling pathway. (H–I) Transcriptional expression levels of Wnt signaling pathway and bone remodeling in human BMSCs derived from hCTR and hB-ALL.


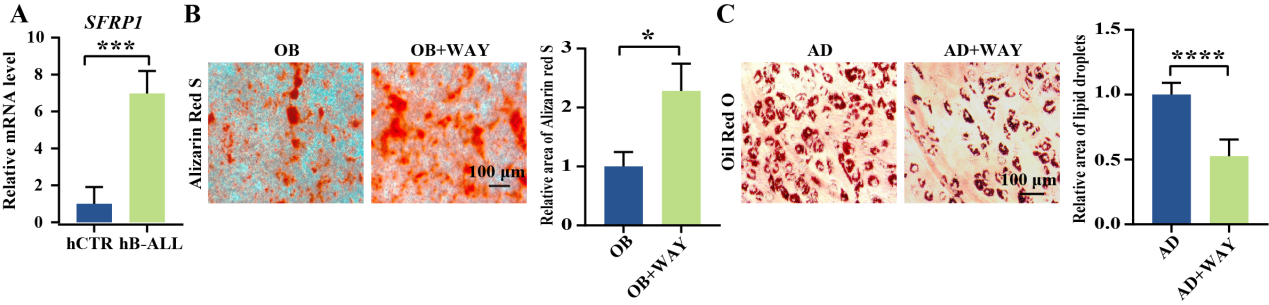


**Supplementary Figure 5.** Effect of SFRP1 on the differentiation of hBMSCs from B-ALL patients. (A) Expression of *SFRP1* gene in hBMSCs from hCTR and hB-ALL patients. (B) ARS staining and quantification of calcium nodule formation after osteogenic induction of hBMSCs from B-ALL patients with or without WAY-316606 treatment. (C) Oil Red O staining and quantification of lipid droplet formation after adipogenic induction of hBMSCs from B-ALL patients with or without WAY-316606 treatment. n=3; ****P* <0.05, ****P* <0.001, *****P* <0.0001.**

**
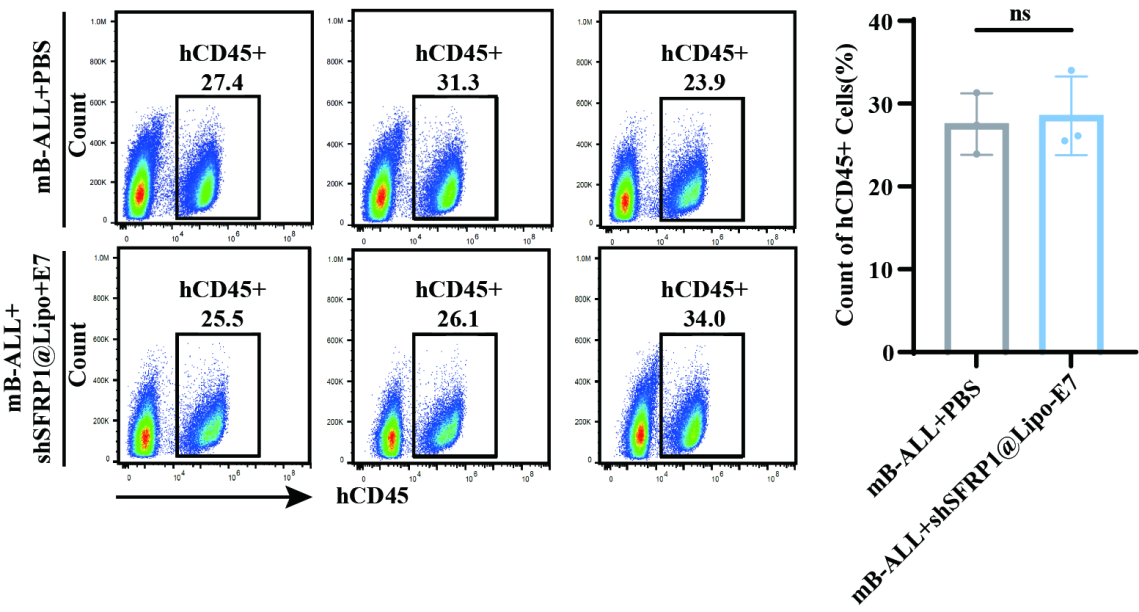
**

**Supplementary Figure 6**. *In vivo* effects on peripheral leukemic cells proliferation of shSFRP1@Lipo-E7.

**
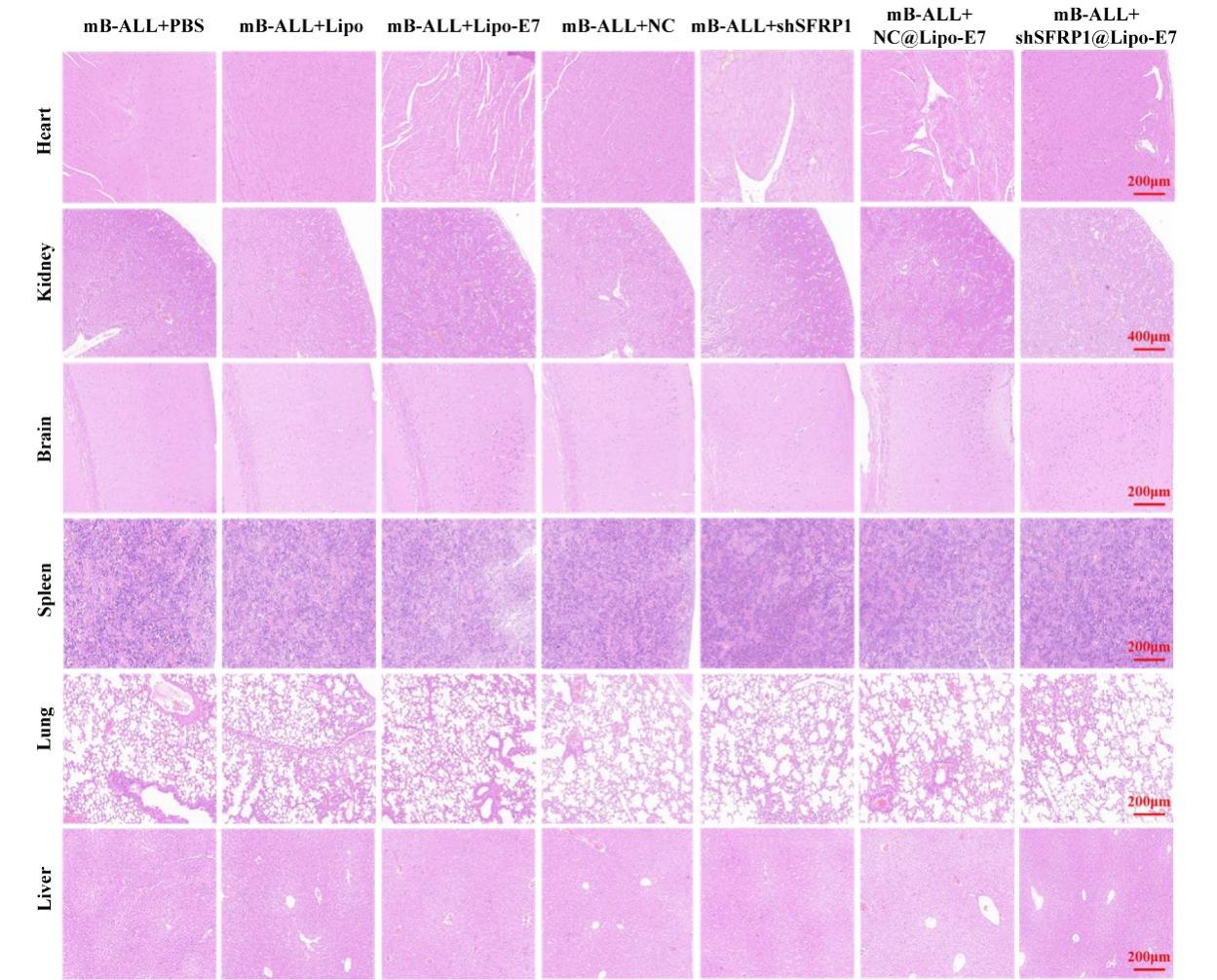
Supplementary Figure 7**. Biosafety evaluation of liposomes by H&E staining of major organs.

**
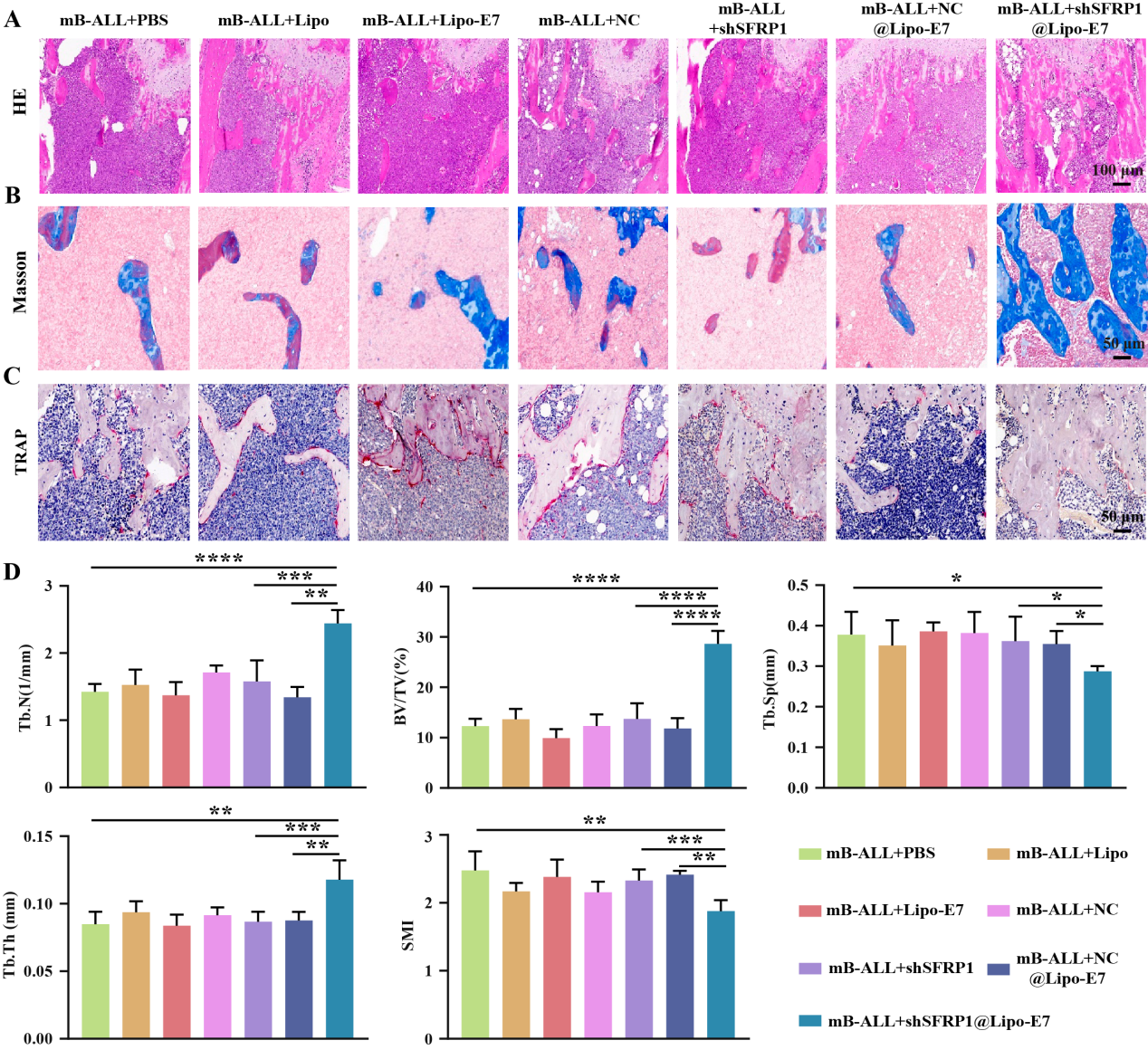
Supplementary Figure 8**. shSFRP1@Lipo-E7 rescues bone loss in B-ALL mice. (A) H&E staining of femurs from B-ALL mice. (B) Masson’s trichrome staining of femurs from B-ALL mice. (C) TRAP staining and quantification of osteoclast surface area per bone surface (Oc.S/BS). (D) Quantitative micro-CT analysis of femoral bone parameters in B-ALL mice. (n = 5; ****P* <0.05, ***P* <0.01, ****P* <0.001, *****P* <0.0001**)

**Supplementary Table 1. Basic information and vertebral CT values between hCTR and hB-ALL groups.**

| **Age (years)** | **Characteristics** | **hCTR** | **hB-ALL** | **P-value** |
| --- | --- | --- | --- | --- |
| 6≤Age＜10 | n | 98 | 95 |  |
|  | Sex Distribution（Male/Female） | 67/31 | 66/29 | 0.751 |
|  | Age,years,(Mean±SD) | 7.77±1.09 | 7.53±1.17 | 0.1467 |
|  | BMI,Kg/m^2^,(Mean±SD) | 16.32±3.08 | 16.08±2.57 | 0.5612 |
|  | Hounsfield Units, (Mean±SD) | 248.5±31.2 | 247.0±32.9 | 0.7493 |
| 10≤Age＜19 | n | 109 | 74 |  |
|  | Sex Distribution（Male/Female） | 70/39 | 50/24 | 0.307 |
|  | Age,years,(Mean±SD) | 12.48±1.55 | 12.05±1.66 | 0.0746 |
|  | BMI,Kg/m^2^,(Mean±SD) | 19.60±4.06 | 18.54±3.82 | 0.0747 |
|  | Hounsfield Units, (Mean±SD) | 260.1±28.2 | 244.4±31.7 | 0.0006 |

**Supplementary Table 2. PCR primer sequences of human genes**

| **Gene name** | **Primers** | **Primer’s sequence (5’-3’)** |
| --- | --- | --- |
| Alkaline phosphatase (ALP) | F | GTGGAGTATGAGAGTGACGAGAAA |
|  | R | CAGATGAAGTGGGAGTGCTTGTA |
| Osteocalcin (OCN) | F | CCACCGAGACACCATGAGAG |
|  | R | CGCCTGGGTCTCTTCACTAC |
| Collagen type Ⅰ α1 chain (COL1A1) | F | CCCAGCCACAAAGAGTCTACAT |
|  | R | GATTGGTGGGATGTCTTCGTCTT |
| Osteoprotegerin (OPN) | F | CATATGATGGCCGAGGTGATAGT |
|  | R | CTTTCCATGTGTGAGGTGATGTC |
| Runt-related transcription factor 2 (RUNX2) | F | AGCAAGGTTCAACGATCTGAGAT |
|  | R | TTCCCGAGGTCCATCTACTGTAA |
| Sex-determining region Y-box 9 protein (SOX9) | F | GACTTCTGAACGAGAGCGAGA |
|  | R | CCGTTCTTCACCGACTTCCTC |
| Collagen type Ⅱ α1 chain (COL2a1) | F | TCAAGTCCCTCAACAACCAGATT |
|  | R | TTCTTGGGAACGTTTGCTGGATT |
| Metallopeptidase 13 (MMP13) | F | GGTCCGATGTAACTCCTCTGAAT |
|  | R | TGGAACTACTTGTCCAGGTTTCA |
| Glyceraldehyde-3-phosphate dehydrogenase (GAPDH) | F | GGAGTCCACTGGCGTCTTCA |
|  | R | GTCATGAGTCCTTCCACGATACC |
| Aspartate-glutamate carrier 1 (AGC1) | F | GAGAGGACTGTGTGGTGATGATC |
|  | R | CGCACCAGGGAATTGATCTCATA |
| Secreted frizzled-related protein 1 (SFRP1) | F | CTTCTACTGGCCCGAGATGC |
|  | R | ATCCTCAGTGCAAACTCGCT |
| Bone morphogenetic protein 2 (BMP2) | F | CCCTACATGCTAGACCTGTATCG |
|  | R | TTTCCCACTCGTTTCTGGTAGTT |

**Supplementary Table 3. Key reagents**

| **Reagent Name** | **Dilution ratio** | **Product Number** | **Manufacturer** |
| --- | --- | --- | --- |
| Anti-ALP antibody | 1:1000 | ab203106 | Abcam, USA |
| Anti-COL1A1 antibody | 1:1000 | ab138492 | Abcam, USA |
| Anti-RUNX2 antibody | 1:1000 | 20700-1-AP | Proteintech, China |
| Anti-SFRP1 antibody | 1:2000 | 26460-1-AP | Proteintech, China |
| Anti-β-catenin antibody | 1:5000 | 17565-1-AP | Proteintech, China |
| Anti-ADIPOQ antibody | 1:1000 | 21613-1-AP | Proteintech, China |
| Anti-SOX9 antibody | 1:1000 | ab185230 | Abcam, USA |
| Anti-Tubulin antibody | 1:5000 | 10094-1-AP | Proteintech, China |
| Anti-GAPDH antibody | 1:50000 | ab181602 | Abcam, USA |

**Supplementary Table 4. Clinical characteristics of bone marrow samples for omics sequencing**

| **Sample ID 1** | **Sample ID 2** | **Age (years)** | **Sex** | **Diagnosis and Classification** |
| --- | --- | --- | --- | --- |
| hCTR1 | C1 | 14.25 | Female | Hematopoietic Stem Cell Donor |
| hCTR 2 | C2 | 12 | Male | Hematopoietic Stem Cell Donor |
| hCTR 3 | C3 | 13.5 | Male | Hematopoietic Stem Cell Donor |
| hCTR 4 | C4 | 13 | Male | Hematopoietic Stem Cell Donor |
| hCTR 5 | C5 | 12.75 | Female | Hematopoietic Stem Cell Donor |
| hB-ALL1 | B1 | 16 | Female | B-ALL/Medium-risk |
| hB-ALL2 | B2 | 12 | Female | B-ALL/Medium-risk |
| hB-ALL3 | B3 | 13.5 | Male | B-ALL/Medium-risk |
